# Patterns of diversity in biomedical coauthorships: An analysis across authors’ ethnicity, gender, age, and expertise

**DOI:** 10.1371/journal.pone.0316890

**Published:** 2025-01-31

**Authors:** Apratim Mishra, Haejin Lee, Sullam Jeoung, Vetle I. Torvik, Jana Diesner

**Affiliations:** 1 School of Information Sciences, University of Illinois at Urbana-Champaign, Champaign, Illinois, United States of America; 2 School of Social Sciences and Technology, Technical University of Munich, Munich, Germany; Institute of Medical Biochemistry Leopoldo de Meis (IBqM) - Federal University of Rio de Janeiro (UFRJ), BRAZIL

## Abstract

Multiple studies have linked diversity in scientific collaborations to innovative and impactful research. Here, we explore how different diversity indices—ethnicity, gender, academic age, and topical expertise—interact and thereby influence scientific impact. Leveraging nearly 900,000 biomedical journal articles from PubMed, published in major journals between 1991 and 2014, we investigate the nuanced relationships among these diversity indices and their collective influence on research outcomes. By systematically varying model parametrizations, we assess the robustness of the observed relationships and examine multiple methodological choices. Our findings reveal a consistent pattern of demographic homophily, where scientists tend to collaborate with others who share similar ethnic and gender backgrounds. While each diversity index correlates significantly with impact when considered individually, gender diversity and topical expertise emerge as the strongest positive predictors of impact after accounting for key covariates. However, the association between diversity and impact is moderated by the number of collaborating authors, with larger teams sometimes showing opposite trends due to interactions between the computed diversity indices and team size. Despite this complexity, the practical drivers of scientific impact for an article remain the journal of publication, authors’ prior citation rate, and the number of co-authors. On further examining expertise diversity through three separate dimensions: variety, balance, and disparity, our findings indicate that impactful teams balance a wide range of subject matter expertise while maintaining a focused connection on closely related topics. These findings highlight the importance of strategic team composition and underline the significance of team diversity in scientific research.

## Introduction

Modern society places significance on the concept of diversity, as evidenced by various scientific articles [1, 2]. Diversity reflecting the differences among individuals, for a range of characteristics or attributes, is recognized for its role in improving economic productivity [3], social cohesion [4], innovation, and creativity [5]. However, the effectiveness of diversity initiatives, particularly those targeting intersections of socio-demographic factors like ethnicity, gender, age, and socio-economic status, remains unclear [5, 6]. It is possible that continually increasing diversity may not positively impact knowledge integration and social engagement [7]. For example, ethnic heterogeneity among groups can create polarization, leading to reduced economic growth and lower levels of economic investment [3, 8]. While some studies in diversity show benefits for mental health and community well-being, if not managed effectively, it can lead to adverse effects such as conflict and feelings of resentment [4, 9]. Highly heterogeneous teams may have different perceptions of the assigned task, creating knowledge boundaries among team members. Furthermore, an individual has multiple facets of characteristics (like gender, ethnicity, class), and the intersectionality of identities is essential to explore as diversity dimensions are interconnected [10]. Thus, diversity can boost creativity and economic benefits, but it also presents challenges, such as discomfort and distrust in interactions across different backgrounds [3, 11, 12].

The practice of scientific collaboration in academia illustrates the diversity in action, as co-authorship reflects the attributes of researchers involved [12, 13]. Researchers come together across disciplines, skills, community backgrounds, and varied institutions to achieve higher-quality results and solve critically important issues. Such collaborative efforts often result in more cited papers (a crude measure of higher impact science) and can also significantly influence societal outcomes and social media visibility [14, 15]. Consequently, many socio-cultural elements in research collaborations have been examined to determine their relative significance and association with scientific impact. Generally, research papers exhibit a positive correlation between the number of collaborating authors and both the article’s impact and the quality of peer review [16, 17]. Previous studies have also indicated that ethnic diversity has the strongest association with scientific impact compared to factors like gender, academic experience, or scientific field [18, 19]. Additionally, researchers have a tendency towards ethnic homophily, indicating that individuals collaborate more with others of the same ethnic background [20]. However, in some fields, ethnically diverse teams may receive fewer citations [21], and ethnic homophily often correlates with lower-impact publications [20]. For gender, productive research teams usually include a mix of genders, with gender diversity being associated with a broader group of research questions being addressed [22–24]. Male researchers are more likely to collaborate with each other and across international borders, while women are disadvantaged in citation networks, especially when holding first or last author positions [25]. The gap in scientific productivity for women further increases with the increase in age, with potential factors including smaller collaborative networks, unfair division of labor, and limited access to funding [26]. The best-performing teams usually have a strong core of authors from the same department or institution, combined with a moderate level of international collaboration [27]. International collaboration tends to enhance the visibility of research articles; however, women being disadvantaged in collaboration networks often leads to receiving fewer citations [25]. At the same time, some countries might contradict this general trend; women researchers in India tend to have more international collaboration [28]. Teams with multiple institutions tend to be more creative, resulting in more significant research regarding the article impact and citation count [29, 30]. The increased global mobility among researchers also contributes to diverse collaborations, mainly due to the presence of elite institutions that drive scientific breakthroughs [31, 32].

### Scientific impact and its relationship with collaboration diversity

Scientific collaborations among researchers present an important domain for studying co-authorship diversity, with citation-based proxies frequently used to measure scientific impact [33]. At the article level, several methodologies have been developed to evaluate scientific impact, including citation percentiles, eigenvector normalizations, and source-normalized article metrics [34–37]. Such approaches reflect the wide range of quantitative metrics used in previous studies but also highlight the lack of standardization in defining a publication’s impact. Commonly used measures, such as the five-year citation window and the Hirsch index (h-index), are often applied to assess the impact of articles, journals, and researchers [18, 38, 39]. When examining the relationship between ethnic diversity in research teams and citation impact, researchers have utilized citation counts over three and six years, as well as considered the total citation count till data retrieval [19, 20]). Some researchers compare citation ratios in relation to the impact factors of journals [40]), while others have opted for field-normalized citation scores [41]. Gender diversity has similarly been studied using raw citation counts [21]. Studies have also adopted different time frames for assessing the long-term impact of citations, with ranges including 13 years, 18 years, 21 years, and some even focusing on citations within the first one or two years after publication [42–44]. The choice of citation window and the use of different citation normalization methods can significantly affect research outcomes. Shorter time windows tend to capture more immediate discourses and visibility [45], whereas citations accumulated over longer periods are more reflective of established knowledge and lasting influence. Despite such variations, all forms of citations are generally regarded as indicators of a publication’s scientific impact [46]. Citation practices also vary by discipline, with each field having its own growth rate, delay, and citation pattern [47]. The timing of when am article reaches its peak citation count can also differ depending on the journal of publication [48]. To address the limitation of traditional metrics, this study uses the relative citation ratio (denoted as *RCR*), a field-normalized and article-level bibliometric assessment of scientific productivity [49]. *RCR* offers a more nuanced approach to citation analysis, accounting for the variations in distinct academic fields through a co-citation network, and is freely available through a web-based tool, iCite [50]. ‘Scientific impact,’ in this study, thus refers to the paper-level citation impact computed using the relative citation ratio.

With this framework, we aim to answer whether diverse author characteristics in collaborative research are linked to a greater scientific impact of publications. Although the above-outlined previous studies have examined the impact of diversity within various research groups, this analysis specifically focuses on biomedical data at the article level, using a snapshot of PubMed, an online database hosted by the U.S. National Library of Medicine. The relative importance of the four diversity indices, ethnicity, gender, academic age, and topical expertise, is evaluated in relation to impact, adjusting for confounders and interaction effects. Using PubMed uniquely allows us to leverage the capabilities of specific “MeSH” keywords (Medical Subject Headings), used to define an author’s “expertise.” MeSH constitutes a controlled and hierarchically organized vocabulary employed in PubMed to categorize research papers, thereby enabling efficient information indexing, retrieval, and classification [51]. Expertise, thus, covers the author-level contribution in an article and is, thus, a temporal quantification of the author based on common concepts (such as diseases, therapies, and procedures) covered in prior papers. Therefore, this study first computes author-level attributes and then calculates an article-level diversity value (diversity index) and their relationship with the article’s impact. The MeSH terms are considerably different from broader classifications like subject categories (in Web of Science) utilized in studies related to the topic of ‘Interdisciplinarity,’ which is an article-level measure generally based on references [52, 53].

The data reveal strong evidence of homophily in ethnicity and gender in research co-authorships, indicating that researchers tend to collaborate with those with similar demographic backgrounds. Using a robust analysis framework that accounts for multiple sources of variation, we utilize generalized linear models to identify the relative importance of diversity indices with scientific impact while adjusting for confounders and interaction effects. For most teams, gender and expertise diversity show a significant and consistent positive association with scientific impact, whereas ethnicity and age diversity show a negative relationship. The interaction between diversity and author count complicates this relationship by acting as a mediating factor, especially at higher levels of diversity. At the same time, the practical significance of the results is influenced by the inclusion of covariates and the interaction between terms, as the strength of the diversity estimates decreases when they are included. Additionally, we explore the abstract nature of expertise diversity through a multidimensional conceptualization, capturing three distinct attributes: variety, balance, and disparity, each representing an essential but individually incomplete aspect of diversity [12, 52, 54, 55].

⋅ *Variety* represents the number of distinct elements contributing to expertise diversity, measured by the unique count of MeSH terms for an article.
⋅ *Balance* reflects the distribution of those elements within expertise diversity, indicating the relative proportions of MeSH terms across the article.
⋅ *Disparity* captures the topical distance between elements, measuring how distant the MeSH terms are from one another.

Our findings show that scientific impact initially declines with increasing variety but rises at higher levels, suggesting that research benefits when collaborators, as a group, have collectively explored a broad range of topics. However, scientific impact tends to decrease with higher levels of balance and disparity, indicating that teams composed of researchers with subject expertise that is too diverse or focused on diverging unrelated areas are less likely to produce highly cited work. Collaborators with a shared, well-defined disciplinary focus in their past research tend to have more citation success. These results suggest that while diversity is valuable, a more cohesive and focused collaboration leads to higher-impact research.

## Results

### Descriptive statistics

This study examines the diversity among co-authors of published articles in terms of four different measures: ethnicity (eth), gender (gen), academic age (age), and expertise (exp), with each measure representing a social factor that can potentially influence collaboration. Here, ethnicity and gender (M, F, and Unknown) indicate an algorithmically predicted value to an author’s name. Academic age refers to the experience a researcher has in terms of the count of prior published papers, and expertise indicates a set of topical keywords that best reflect a researcher’s specific knowledge and skills within PubMed. Table 1 presents the descriptive statistics and correlation values between the different diversity indices, *d*_*x*_ ∈ (eth, gen, age, exp). The results show no substantial correlation between them, indicating the absence of a significant linear relationship, with minor correlations likely due to the rise in diversity as the number of authors increases. The distribution of expertise (exp) is right-skewed, so a logarithmic transformation (denoted as *Log*.*d*_*exp*_) is applied to normalize the distribution and improve the reliability of statistical modeling. Taking this into account, we proceed with further testing and regression analysis to understand the relationship between diversity and scientific impact.

**Table 1:** Descriptive statistics for all diversity indices (Normalized).

Descriptive Statistics (Normalized)						Correlation value between diversity indices				
	Avg.	Std. Dev	Median	Min.	Max.		*d*_*eth*_	*d*_*gen*_	*d*_*age*_	*d*_*exp*_
*d*_*eth*_	0.455	0.294	0.533	0.0	1.0	*d*_*eth*_	1.0			
*d*_*gen*_	0.437	0.275	0.544	0.0	1.0	*d*_*gen*_	0.136	1.0		
*d*_*age*_	0.464	0.182	0.479	0.0	1.0	*d*_*age*_	0.079	0.141	1.0	
*d*_*exp*_	0.232	0.16	0.188	0.0	1.0	*d*_*exp*_	0.17	0.173	0.005	1.0

### Examining patterns of homophily

**Fig 1:**
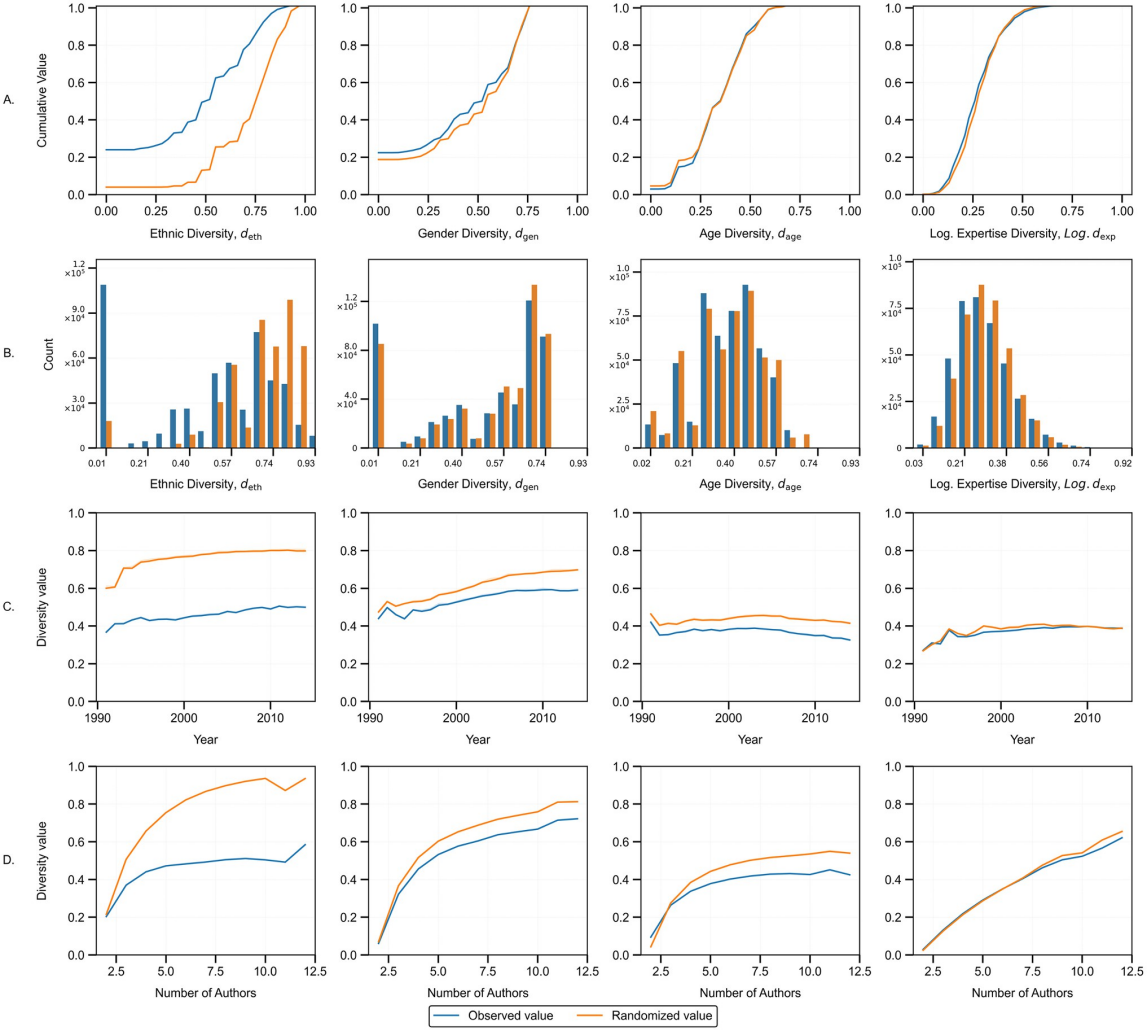
Examining homophily for each diversity index by comparing the observed data with the randomized data. Each column corresponds to a diversity index, *d*_*x*_:*x* ∈ (eth, gen, age, Log. exp), and each row compares the observed to the randomized data based on a specific experiment. A: Cumulative distribution of *d*_*x*_. B: Count values of *d*_*x*_ across the span of possible values. C: Change in mean diversity index value over time. D: Change in mean diversity index value over author count.

To begin our analysis, we first investigate the presence of homophily in author collaborations, focusing on whether researchers tend to collaborate more frequently with individuals who share similar characteristics. The process involves generating randomly shuffled datasets for each diversity index across all considered articles, conditioned on publication years and author counts. Repeating this shuffling process multiple times allows for calculating the diversity distribution under random conditions, thereby enabling a comparison with observed trends (See Note 2.3 in S1 File for details defining this process). Fig 1 compares the observed data value to the randomized data distribution for each diversity index, *d*_*x*_:*x* ∈ (eth, gen, age, Log. exp). The first row, Fig 1A, indicates the cumulative distribution for each diversity index, revealing pronounced homophily with respect to ethnic diversity, to a moderate extent in gender diversity, and no significant patterns in age and expertise. This indicates that researchers tend to collaborate with others of similar ethnic and gender backgrounds than would occur by chance. The temporal analysis, reflected in Fig 1C, indicates that both real *d*_*gen*_ and *d*_*eth*_ have increased over the period of the dataset. This finding suggests a growing inclusion of researchers from a wider range of ethnic backgrounds as well as a gradual increase in female participation in research. Finally, team size correlates positively with diversity across all indices, indicating a potential interaction between diversity and author count. Lastly, papers with a lower author count(two or three) often display higher-than-expected age diversity, likely reflecting collaborations between junior students and more experienced researchers.

### Relationship between diversity and scientific impact

**Fig 2:**
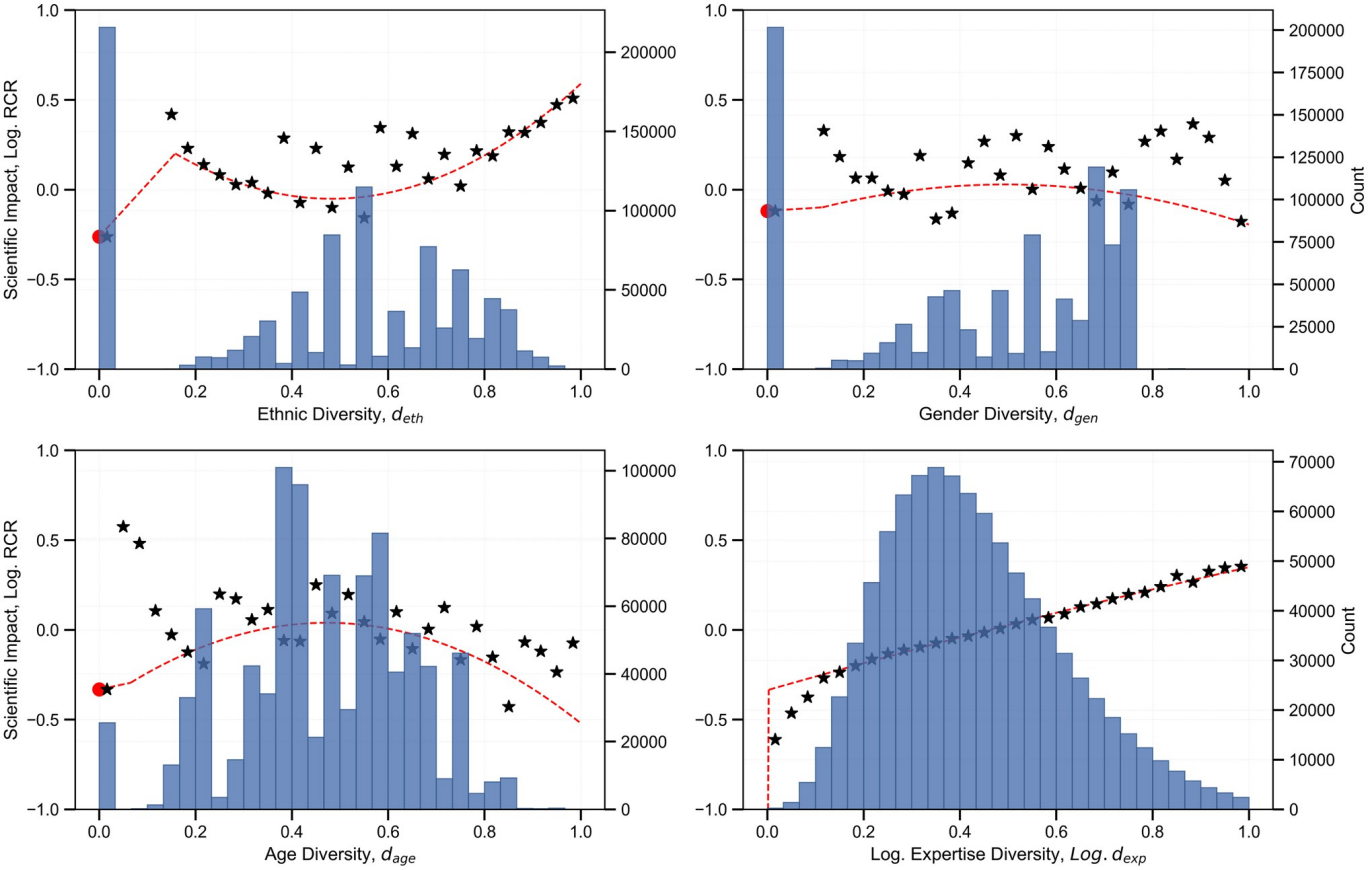
Regression fit between each diversity index and the article’s scientific impact, including a dummy variable. Each curve is overlaid on a histogram of the diversity distribution, with an asterisk (*) marking the mean for each bin. The y-axis indicates the logarithmic value of *RCR* as used in the final model.

While homophily is observed for *d*_*eth*_ and *d*_*gen*_, a linear model cannot be confirmed to capture the optimal fit. Subsequently, as plotted in Fig 2, a quadratic term with a dummy variable captures non-linearities and facilitates a better fit to the data. A higher adjusted R-squared for the quadratic model, and a significant second-order coefficient confirm the superior fit for each diversity index (Table B in the S1 File confirms the improved fit for the higher-order model). Additionally, a positive and statistically significant correlation is observed between the mean grouped diversity value (grouped on author count and year of publication) and scientific impact (see S1 Fig). However, the range of association varying between 0.48—0.72 indicates that the relationship between scientific impact and the diversity indices could be of a higher order. Also, S2 Fig highlights the interaction between the diversity indices and author count, underscoring the importance of author count as a crucial variable in our regression model. By controlling for author count, we account for the differences in collaborative scale and are able to capture team-size relationships. The line plots for different curves based on varying author counts reveal the need to account for author interactions and ensure that the relationship with impact is not biased or inflated. Furthermore, papers are segmented by journal domains: biology, medicine, and science to obtain more structured insights among broad subject categories in PubMed as evidenced by a previous study [56] (See Table A in S1 File to view journal distribution in the dataset).

**Fig 3:**
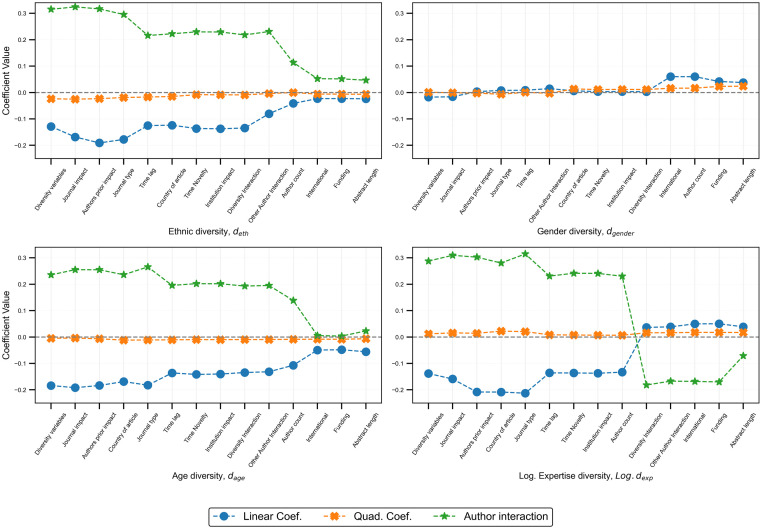
Change in coefficient of diversity indices at each model-fitting step. Each subplot shows the contribution of the specific diversity index (linear, quadratic, and author interaction) at each iterative model fitting process, post-evaluating the best combination of variables. In all, confounding factors minimize the effect size of the diversity index and the diversity-author interaction.

Considering the right-skewed distribution of the dependent variable (*RCR*), a Tweedie regression model with a natural logarithm link function is applied to appropriately capture the relationship between impact and the independent variables [57]. The Tweedie model, suited for data with a mix of zero and positive values, is fitted using the ideal power parameter (1.95), minimizing the Akaike Information Criterion (AIC) for optimal fit. This choice is supported by the observed heteroscedasticity and non-normal residuals, which violate ordinary least squares (OLS) regression assumptions. The variance inflation factor (VIF) less than 5 indicates that multicollinearity is not a concern, meaning that the model can accurately estimate regression coefficients. Additionally, the following variables known to be related to citations from previous studies were incorporated as controls—author counts, time since publication, abstract length, institutional impact, authors’ prior impact, paper novelty, journal impact, funding presence, and international collaboration [58–60] (Table C in S1 File justifies the added covariates improving the model fit, and Table D in S1 File explains the optimal order of the added covariates). The low correlation between the independent variables supports their inclusion in the regression model (presented in Table E in S1 File). Table 2 presents the regression results for both the simple models, where scientific impact is regressed individually against each diversity index, and the full model includes all covariates. In the simple models, all diversity indices are statistically significant, with relatively larger effect sizes. However, when controlling for additional factors in the full model, we observe significant coefficient changes, suggesting potential confounding effects. Our analysis reveals that while all diversity indices are statistically significant, their effect sizes vary depending on the model’s included factors (see Fig 3). The regression is justified by the fits shown in S3 Fig, and the effect sizes capture the multiplicative effects of the diversity indices and the confounding variables on scientific impact.

**Table 2:** Regression analysis of diversity indices on scientific impact (*RCR*).

Variables	Simple Model ª		Complete Model º	
	Coef.	Std Err	Coef.	Std Err
Constant			0.4898	0.005**
Time lag	0.035**	0.002	0.1278**	0.002
Author count	0.149**	0.002	0.12**	0.007
Prior citation rate	0.122**	0.002	0.0964**	0.002
Abstract length	0.067**	0.002	0.1275**	0.002
Abstract length^2^	-0.003*	0.001	-0.0141**	0.001
Institution impact	0.198**	0.002	0.0469**	0.002
Institution impact^2^	-0.025*	0.001	-0.0028**	0.001
Paper Novelty	-0.022**	0.002	-0.0036*	0.002
Paper Novelty^2^	0.058**	0.002	0.0397**	0.001
Journal impact	0.382*	0.001	0.3257**	0.002
Journal impact^2^	0.082**	0.002	0.1089**	0.001
Journal impact^3^	-0.002*	0.001	0.0217**	0.001
International collab.	0.100**	0.002	0.0125**	0.003
Funding	0.125**	0.002	0.0669**	0.004
**Diversity indices**				
*d*_*eth*_	0.124**	0.002	-0.0258**	0.005
$d_{eth}^{2}$	-0.013**	0.002	-0.0058**	0.002
*d*_*gen*_	-0.008**	0.002	0.0372**	0.006
$d_{gen}^{2}$	-0.016**	0.002	0.0249**	0.002
*d*_*age*_	-0.041**	0.002	-0.0627**	0.004
$d_{age}^{2}$	-0.03**	0.001	-0.0069**	0.001
log *d*_*exp*_	0.069**	0.002	0.0136**	0.005
(log *d*_*exp*_)^2^	0.013**	0.001	0.0192**	0.001
Interaction terms				
*d*_*eth*_ × *d*_*gen*_			-0.0122**	0.004
*d*_*eth*_ × log *d*_*exp*_			0.0037	0.006
*d*_*gen*_ × *d*_*age*_			0.0125**	0.004
*d*_*gen*_ × log *d*_*exp*_			-0.049**	0.005
*d*_*eth*_ × Author count			0.0569**	0.005
*d*_*age*_ × Author count			0.0231**	0.006
log *d*_*exp*_ × Author count			-0.0714**	0.008
**Fixed Effects**				
Country of publication			Included	
Journal type			Included	

Standard Error in parenthesis:

** *p* < 0.01;

* *p* < 0.05;

N = 907024

This regression table presents the relationship between the four diversity indices, *d*_*x*_ ∈ (*eth*, *gen*, *age*, *exp*), against scientific impact, *RCR*. The first column indicates the simple model (ª) where only the singular independent variable is used for modeling (GLM with logarithm link). Next, complete models are presented, incorporating all confounding variables.

Ethnic diversity shows a positive coefficient in the simple model (0.124) but shifts to a negative association (-0.0258) in the full model. This significant change suggests that, while ethnic diversity initially appears to boost impact, the inclusion of control variables reveals a negative relationship. The quadratic term for ethnic diversity follows a similar pattern, decreasing from -0.013 to -0.0058, indicating a stronger diminishing return when additional factors are considered. However, a significant positive interaction with the author count in the final model (coefficient: 0.0569) suggests that larger research teams (greater than the median team size of 5) would benefit from greater ethnic diversity. Similarly, gender diversity presents a slight negative coefficient in the simple model (-0.008), but in the full model, this shifts to a positive and significant coefficient (0.0372). The quadratic term for gender diversity also reverses, from -0.016 to 0.0249, highlighting that the positive impact of gender diversity grows as the diversity value increases. Interestingly, while combining ethnic and gender diversity tends to reduce impact (coefficient: -0.0122), larger author teams mitigate this estimate in terms of practical significance, suggesting that team size plays a key role in effect size. Age diversity shows a consistent negative association across both models, though the magnitude intensifies in the full model (-0.041 to -0.0627), suggesting that as team members’ academic ages diverge, the negative impact on scientific outcomes becomes more pronounced when other factors are controlled. However, the positive interaction with gender diversity indicates that mixed-gender teams with junior and senior researchers yield better research outcomes. Expertise diversity, on a logarithmic scale, shows a positive relationship with scientific impact (coefficient: 0.0136), with this effect size increasing at higher levels (positive quadratic term of 0.0192), highlighting the value of team members with complementary knowledge backgrounds. However, a negative interaction of expertise diversity with author count reveals that this benefit is most pronounced in smaller and medium-scale teams. As the author count increases, there exists a diminishing result due to the interaction effect, suggesting that while diversity is advantageous, larger teams may encounter challenges such as conflicts or inefficiencies, which can reduce their overall scientific impact.

Beyond the explored diversity indices, our analysis reveals the other key factors driving scientific impact. The journal’s impact emerges as the strongest predictor, showing a non-linear positive relationship with scientific impact, suggesting that higher-impact journals increase influence, but this association might taper off, suggesting that other factors become more important in determining scientific impact at the highest levels. The other positively correlated variables with impact include the authors’ prior citation count and the author count, indicating that established researchers and larger collaborative teams produce more influential work, also evidenced by prior studies [61, 62]. Abstract length demonstrates a curvilinear relationship with impact, suggesting an optimal length for maximizing visibility and citations. The length of an abstract has a non-linear relationship with impact, indicating an ideal length for maximizing visibility and citations. International collaboration and funding have modest yet significant positive effects, highlighting the importance of diverse partnerships and financial backing in improving research outcomes [27]. Scientific impact also increases with time since publication and institutional prestige, though the latter also follows a slight non-linear trend. This relationship could imply that extremely prestigious institutions could publish a larger volume of research, some of which may not necessarily achieve a high impact compared to their other work. Novel research follows a non-linear relationship with impact, where highly novel papers may struggle initially to gain recognition but tend to accumulate more citations as time passes. The findings further highlight the multifaceted nature of scientific impact, emphasizing the interplay between researcher attributes, institutional prestige, collaboration patterns, and publication strategies in shaping the influence of scientific work.

### Robustness tests

To ensure the robustness and validity of the results, we conduct a specification curve analysis, evaluating model results across 81 plausible specifications [63]. This methodological approach tests for a comprehensive range of reasonable specifications- those that enable sensible testing of the research question, are expected to be statistically valid, and avoid redundancy. Additionally, this analysis is complemented by an inferential component combining all specifications into a joint statistical test, assessing the combined evidence supporting the estimate of interest. The model choices evaluated are detailed in Table J of the S1 File, and a comprehensive process description is provided in Note 2.5 of the S1 File.

The results show consistent patterns, with stable median effect sizes across specifications, and highlight the necessity of including author count to avoid biased outcomes (S4 Fig illustrates the marginal ordered effect across all specifications). Additionally, the robustness is further assessed by comparing the results to a shuffled null distribution, where the diversity index values were randomized. This process simulates what the findings would look like if no true effect existed. By contrasting the observed specification curve (showing actual effect sizes) with the null distribution, we can determine whether the observed effects are genuine or merely due to random variation. Our findings indicate that gender and expertise diversity have a positive, consistent impact on scientific outcomes, while ethnicity and age diversity tend to show negative relationships. Table 3 summarizes our key tests, comparing the observed effects to the null hypothesis of no effect (S5 Fig illustrates the observed and the under-the-null curve for each diversity index across specifications).

**Table 3:** Joint tests for specification curves for all diversity indices.

Diversity Coeff.	Median Effect Size		Share of significant results		Aggregation of all P values	
	Observed result	P value	Observed result	P value	Stouffer Z	P value
*d*_*eth*_	-0.0374	P < 0.02	75/81 specifications	P < 0.02	26.02	P < 0.02
*d*_*gen*_	0.087	P < 0.02	27/27 specifications	P < 0.02	34.77	P < 0.02
*d*_*age*_	-0.0783	P < 0.02	81/81 specifications	P < 0.02	35.75	P < 0.02
*Log*.*d*_*exp*_	0.0518	P < 0.02	81/81 specifications	P < 0.02	29.94	P < 0.02

Median Effect Size: This column shows the median estimate of the observed diversity index across all model specifications. A positive effect size implies an increase in the dependent variable (scientific impact) with a higher diversity value, while a negative effect size implies a decrease. A low p-value indicates all shuffled results are on one side of the observed value.

Share of Significant Results: This represents the number of specifications that yielded statistically significant results out of the total specifications tested. For example, 75/81 for ethnic diversity indicates that 75 out of 81 specifications yielded a significant estimate. A low p-value indicates fewer significant results under the null (when shuffled).

Aggregation of P-Values: Stouffer’s Z [64] is computed by converting each P-value to a Z-score and taking a weighted average, with weights determined by the number of tests (weighted by the inverse square root). This aggregation considers the lack of independence across specifications, and presents a combined measure of significance across all models. The P-value here is obtained by resampling (shuffling) rather than relying on a standard normal distribution, accounting for correlated specifications.

P-Values: P-values indicate the proportion of shuffled samples with test statistics as extreme as the observed data. When no shuffled sample is as extreme, we report *P* < 0.02 for two-tailed tests, indicating a robust relationship unlikely to have occurred by chance.

### Multidimensional conceptualization of expertise diversity

Considering the significant and positive relationship of expertise diversity with scientific impact, it is important to present a fine-grained analysis of this measure of diversity. Thus, here we study expertise diversity as a diversity of categories consisting of three separate dimensions: variety, balance, and disparity [12, 55]. Table 4 confirms that the correlations among the three attributes, variety, balance, and disparity, are not significant, indicating distinct attributes of expertise diversity, *d*_*exp*_. Specifically, we observe negative correlations between variety and balance (-0.319), between balance and disparity (-0.278), and a positive correlation between variety and disparity (0.058). This confirms that the three attributes reflect distinct properties of expertise diversity, *d*_*exp*_, and provide evidence for examining their individual relationship with scientific impact.

**Table 4:** Multidimensional attributes of *d*_*exp*_: Variety, balance and disparity.

Descriptive Statistics (Normalized)						Correlation value between diversity indices				
	Avg.	Std.Dev	Median	Min	Max		*d*_*exp*_	*d*_*variety*_	*d*_*balance*_	*d*_*disparity*_
*d*_*exp*_	0.232	0.16	0.188	0.0	1.0	*d*_*exp*_	1.0			
*d*_*variety*_	0.306	0.119	0.292	0.0	1.0	*d*_*variety*_	0.26	1.0		
*d*_*balance*_	0.7	0.115	0.707	0.0	1.0	*d*_*balance*_	-0.52	-0.319	1.0	
*d*_*disparity*_	0.316	0.156	0.285	0.0	1.0	*d*_*disparity*_	0.829	0.058	-0.278	1.0

**Fig 4:**
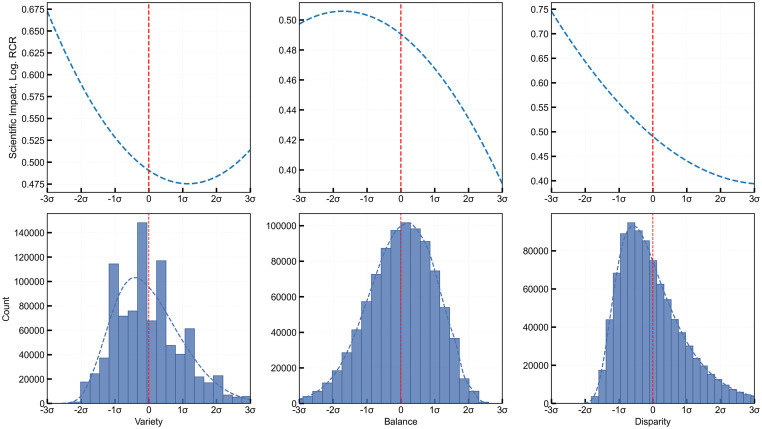
Regression fit between expertise diversity attributes and scientific impact. (a) Second-order regression fit of standardized expertise diversity attributes—variety, balance, and disparity against scientific impact (*RCR*). (b) Distribution of articles over variety, balance, and disparity, highlighting an initial decline then increase in impact with variety, while balance and disparity mostly reduce impact.

Table F in S1 File presents the results of the complete regression model that includes all possible confounders. Utilizing a Tweedie regression model (with a logarithmic link function), we observe a U-shaped relationship with variety initially reducing impact (-0.026) but eventually enhancing it (0.011) as the range of topics widens. This pattern suggests that adding more topics may introduce complexities leading to lower article impact; however, as the number of topics increases, with additional authors who bring a broader range of topics, the article’s relevance could improve, enhancing its impact. Conversely, both balance (first order: -0.017 and second order: -0.005) and disparity (first order: -0.059 and second order: 0.009) consistently show a negative association with scientific impact. Therefore, articles with a balanced spread of topics or too distant in the MeSH tree generally see reduced impact. Most articles would benefit from lower disparity values, as a higher disparity suggests that authors from highly diverse fields struggle with communication and methodological alignment, thus lowering the article’s overall impact. However, the negative relationship between disparity and impact weakens beyond a certain threshold (positive second order: 0.009) as the marginal negative estimate gradually decreases. This trend indicates that the relationship between disparity and impact might turn positive, but the threshold is about four standard deviations from the mean and is only reached for a minority of articles. The curvilinear relationships between the three expertise diversity attributes and scientific impact are illustrated in Fig 4.

## Discussion

### Main findings

This study investigates how different types of diversity in author collaborations are associated with scientific impact in PubMed data. We first establish homophily in author collaboration in the case of ethnicity, *d*_*eth*_ and gender, *d*_*gen*_, implying that researchers tend to associate with other individuals of the same ethnicity and gender. Moreover, overall gender and ethnic diversity in research teams has increased as the researcher pool has become more diverse. Since research collaborations are a form of social interaction, it is essential to include adequate covariates to explain their impact. Examining diversity in isolation, without proper controls, can lead to inflated or incorrect estimates. The results indicate that interactions between diversity indices and significant covariates (such as author count) are crucial for accurately identifying their true relationship with scientific impact.

Our analysis shows that gender and expertise diversity enhance scientific impact for most publications. The positive relationship between gender diversity and scientific impact suggests that greater representation of women likely contributes to improved creativity and team dynamics, leading to improved article impact. Similarly, expertise diversity provides a broader range of knowledge (conceptual MeSH terms) and leads to impactful publications. However, the diminishing association of expertise diversity in larger teams (with six or more authors), reflected in its negative interaction with author count, highlights potential coordination and communication challenges in managing diverse expertise at scale. This suggests that while team gender and expertise diversity drive impactful papers, their benefits may plateau as the author count increases. Larger teams may struggle to fully leverage diverse expertise, suggesting that novelty from expertise diversity doesn’t always translate into high-impact papers in larger collaborations. Ethnic and age diversity show more complex effects, depicting a negative relationship that could reflect systemic biases or challenges in integrating diverse cultural backgrounds and age gaps. However, the positive interaction between ethnic diversity and author count suggests that larger teams (with seven or more authors) may better manage and leverage ethnic diversity, likely due to more resources or stronger support structures. While all diversity indices are statistically significant in the final regression model, the primary practical drivers of scientific impact are high-impact journals, larger teams, authors’ prior citations, and affiliations with prominent institutions. Established researchers in well-resourced teams consistently produce more influential work. Some studies have linked collaboration diversity with improved collective decision-making, enhanced team creativity, and even greater brain synchronization [65]. However, other research reports little to no positive relationship between demographic diversity and team performance [66]. Our findings show that while diversity matters, it has a modest correlation with overall impact.

Lastly, an important contribution of this research is the operationalization of expertise diversity through three distinct attributes: variety, balance, and disparity. Variety, defined by the number of unique MeSH subcategories, initially has a negative association with impact but reverses this relationship once a certain threshold is surpassed, likely due to the integration of diverse perspectives. Balance, representing the spread of expertise (distribution of MeSH terms across all authors), consistently has a negative relationship with impact, especially at higher values. This suggests that an uneven spread of varying knowledge and skills in co-authors across too many areas can hinder effective collaboration, likely due to coordination difficulties or a lack of deep focus on key topics. Disparity, or the cognitive distance between MeSH terms, begins with a negative association with scientific impact but stabilizes at higher values. This trend indicates that while too much distance between collaborators regarding knowledge concepts may impede cohesion, a moderate level of disparity can bring fresh insights into research. Together, the results suggest that high-impact publications benefit from a delicate balance of expertise diversity: teams should engage collaborators with a broad range of expertise (high variety), but concentrate on a few core disciplines (low balance) and maintain a low to moderate cognitive distance (disparity).

### Conclusion

Our findings demonstrate that diversity in research teams spanning expertise, gender, ethnicity, and age uniquely shapes scientific impact in complex ways. Here, we align with perspectives from social epistemology that knowledge production is social, where team composition plays a crucial role in research outcomes [67]. Groups with varied backgrounds and training lead to better data processing and decision-making, a concept studied as cognitive diversity [68]. However, while we examined demographic diversity (ethnicity, gender, age) and diversity in knowledge (expertise), other dimensions of cognitive diversity, such as methodological preferences and personality influences, could also be beneficial toward scientific impact.

The results indicate the positive impact of expertise diversity, aligning with arguments that teams with diverse knowledge bases and thinking styles often outperform more homogeneous groups in addressing complex problems. When researchers from various specialties collaborate, they contribute distinct analytical tools and knowledge bases, and tasks are essentially distributed extending beyond individuals [68, 69]. The positive association between gender diversity and scientific impact aligns with epistemological frameworks that advocate for integrating diverse viewpoints, suggesting that social positions bring necessary perspectives to the table [70]. Diverse teams may identify different research questions or interpret data through various theoretical perspectives. Furthermore, the positive outcomes associated with ethnic and age diversity in larger teams imply that well-resourced teams can more effectively distribute ‘cognitive tasks’ [69]. Our analysis of team size and collaboration patterns highlights how diverse methods and knowledge bases within research networks help avoid incorrect or suboptimal conclusions [71]. This exchange appears more effective in larger, well-structured teams, where varied perspectives are more readily managed and integrated. For instance, researchers from different disciplines may conceptualize the same problem through distinct theoretical lenses, employ varied methodological approaches, or draw insights from different bodies of knowledge [4]. Our findings on expertise diversity attributes show that successful collaboration depends not just on assembling cognitively diverse teams but on creating conditions where different thinking styles can effectively combine. These results have practical implications for research practices. We propose that teams enhance cognitive diversity by including members with different backgrounds, problem-solving approaches, and analytical frameworks. Institutions can support this through integrating diverse perspectives, and team leaders can adopt strategies to maximize their impact. Future research could explore how new communication patterns relate to integration [70, 71]. Overall, our study shows that fostering diversity with the right structures is the key to improve research impact and advance scientific knowledge [68].

### Limitations

This study investigates whether diversity within scientific teams can positively influence the impact of their publications. While the results are consistent across a set of model specifications, the results should be taken with caution, given various limitations. First, our data was sampled for three broad areas (biology, medicine, and science), represented by the top 40 most frequent journals in PubMed, with 2–12 authors. Although this selection offers robust and accessible data on authorship and publications, PubMed primarily aggregates articles from biomedical and life sciences journals (e.g., Nature, PNAS, Science). As a result, the findings may not extend to disciplines outside biomedicine, health, or life sciences. Next, to ensure the robustness of our results, we employed specification curve analysis, testing our model findings across multiple parametrizations. However, some researchers may consider certain specifications superior to others. Furthermore, while our analysis encompasses numerous valid specifications, it cannot exhaustively capture every possible analytical approach. Thus, this analysis reduces analytical ambiguity but cannot eliminate it.

Next, we used the Rao-Stirling index to measure diversity, but this is just one option among several viable alternatives, each with its strengths [72–74]. The gender classification tool Genni [76] accounts for the complexities of predicting gender from English names, but it may misclassify many Asian names as “unknown/unisex.” This suggests a potential link between predicted gender and ethnicity, especially given the unique nature of the categories in our dataset (presented in Table H in S1 File). Also, the ethnicity prediction tool, Ethnea [76], categorizes authors into a limited number of groups, which may not fully capture the complexity of ethnicity as a broad concept encompassing shared cultural practices. For example, two authors classified as ‘German’ may have very different cultural backgrounds—one might be an American of German descent, while the other could be a resident of Germany. Additionally, the tool does not recognize ‘African’ as a distinct ethnicity. Another limitation is that Ethnea treats all ethnic categories as equally distant. For instance, it assumes that the distance between German and French is the same as between German and Indian, which may not accurately reflect cultural similarities and differences. Similarly, our understanding of expertise diversity relies on MeSH categories, which may miss important nuances present in finer sub-categories specific to PubMed.

Our scientific impact metric, the Relative Citation Ratio (*RCR*), also differs from other approaches that use citation normalizations or total citation counts over varying periods, potentially leading to variations in the trends and outcomes observed [38, 42]. Citations are only one measure of research success, and various social factors make attributing specific effects to each variable challenging. Other impact metrics, such as mentions in white papers, policy documents, technical reports, or social media, may provide a different understanding of research influence [77]. Finally, while our analysis focuses on the diversity within individual papers, a more accurate approach might involve examining diversity at the research group level, which could offer a better unit of analysis for social dynamics. Researchers increasingly seek strategic collaborations to enhance their impact amid competitive bibliometric pressures [78]. Our study reveals how team dynamics influence scientific impact while highlighting the need to examine further individual integration within research teams. We recommend further exploring collaboration patterns using established measures and qualitative analysis for stronger comparative evidence.

## Data and methods

### Dataset

This research uses a snapshot of PubMed data, including articles in biomedicine and life sciences. To obtain author-disambiguated data, we utilize ‘Authority 2018’ [79], which enables author identification across publications. Disambiguation helps to 1) identify the same author even when the author has published under multiple name variant name, 2) model distinct researcher’s history, and 3) identify author-level data, such as their gender, ethnicity, prior citation count, and computing the diversity indices. Additionally, we incorporate the MapAffil dataset [80] to obtain author-disambiguated affiliation data, including institutional, state, and counter information. Moreover, before December 2013, PubMed did not comprehensively record author affiliation and characteristics for every contributor to a publication. Consequently, the author-disambiguated dataset enables the prediction of reliable imputed information that is author-specific. To identify the relative scientific contribution of a journal, we utilize Scimagojr [81], a publicly available online platform to access the journals’ h-indices. To construct the dataset, we first collected the top 40 journals from the entirety of the dataset. The journals collected were divided into three subject categories: medicine, biology, and science as per previous literature [82]. Next, to get a representative dataset, we sampled papers from the collected journals where the author count was between 2 and 12 (inclusive) between 1991 and 2014 (inclusive). Our analysis spans multiple publication years, allowing articles sufficient time to accumulate citations and ensure robust citation-based analysis [83]. Articles with a single author are not included as they would be assigned a ‘null’ value for diversity. We restricted our study to articles categorized as ‘journal articles,’ excluding review letters, letters to the editor, and news articles. Overall, we analyze 907024 unique papers and 1316838 unique authors.

### Measures

The study uses the Relative Citation Ratio (*RCR*) as the primary variable of interest, obtained from the iCite web application [50], providing bibliometric data for individual scientific publications. Since *RCR* exhibits a right-skewed distribution, we apply a natural logarithm transformation in the modeling process. Utilizing a logarithmic link function, the coefficients reflect the proportional change in *RCR* rather than absolute changes. The control variables are intended to represent author-specific, paper-specific, and journal-specific factors that could influence the number of citations an article receives. The features used for modeling are represented in Table 5.

**Table 5:** Description of all explanatory features.

Feature	Description
Ethnicity	is the author’s ethnicity, predicted using “Ethnea,” a nearest-neighbor algorithm to map an ethnicity to the author’s name. Each author is mapped to one of 20 ethnicities, including an ‘OTHER’ mapping [76].
Gender	is the author’s gender, predicted using Genni + Ethnea, which covers names worldwide and is also sensitive to ethnic variations in names globally. Each author is labeled as one of the following: Female, Male, or Unknown [75, 76].
Age	is the author’s academic age, calculated by counting the number of publications before the year of the current article’s publication, followed by an ordinal classification.
Expertise	is the author’s expertise, defined using a collection of the most common MeSH terms for each author over all previously published articles. For an article, *p*, let all authors be represented as Authors(*p*). Now, for any author, *a*_*i*_ ∈ Authors(*p*), for that article, let MeSH(*a*_*ip*_) denote the aggregated set of MeSH words for that particular author up to the certain article, *p*. Therefore, for the article, *p*, we define “expertise” as an aggregated list: $\begin{array}{cc} \text{expertise}\left(p\right)=\left[\text{MeSH}\left(a_{ip}\right):a_{i}\in \text{Authors}\left(p\right)\right] & \left(1\right) \end{array}$
Time lag	indicates the difference in years between the year of publication and the year 2023.
Author count	is the collaboration size of researchers for the article.
Prior citation rate	is the mean of the authors’ citation rate up to the article’s publication year.
Abstract length	is the length of the abstract for that article.
Institution Impact	is the maximum of each author’s institution publication rate.
Paper Novelty	is a quantifiable measure of an article’s novelty based on the newness of concepts. Here, this measure indicates the ‘Time novelty’ of an article, capturing an article’s originality, and is represented by MeSH terms [82].
Journal Impact	is the impact of the article’s accepted journal of publication, given by Scimago Journal Ranking, which indicates the journal’s h index [81].
International collab.	is a binary indicator referring to the absence of international collaboration for the article’s authors.
Funding	is a binary indicator referring to the presence or absence of any author receiving any funding for the article’s year of publication.
Country of publication	is a fixed effect for the article’s country, as inferred by MapAffil [84].
Journal type	is a fixed effect modeled as a dummy indicator for the journal type against the type ‘medicine.’

#### Diversity measures

Our analysis explores the dynamics of collaboration diversity among researchers regarding the authors’ gender, ethnicity, academic age, and expertise. Functional diversity is a widely studied research topic in biological communities to examine ecosystem processes [85, 86]. To compute *d*_*gen*_, *d*_*age*_ and *d*_*exp*_, we employ the Rao-Stirling index (*Q*), which uses the abundance of entities and their pairwise distance to compute diversity [55, 87]. For *d*_*gen*_, ‘Male’ and ‘Female’ are assigned a unit distance, while authors labeled ‘unknown/unisex’ are positioned at a half-unit distance from the other categories. For *d*_*age*_, the numerical age value is categorized into discrete bins, facilitating the calculation of co-authors’ age diversity value. Rao-Stirling index (*Q*) for any article, *p* is computed using Eq 2, where *S* is the total number of diversity elements. The term *d*_*kl*_ quantifies the distance, and *P*_*k*_ and *P*_*l*_ denote the proportions of individual categories, *k* and *l*.

$$\begin{array}{cc} & Q\left(p\right)=\sum_{k=1}^{S}\sum_{l=1}^{S}d_{kl}P_{k}P_{l} \end{array}$$ (2)

To further understand expertise diversity, we adopt a multidimensional approach of decomposing this diversity index into three attributes: variety, balance, and disparity [55, 87]. Let *p* denote a research article in our dataset. For each author *i* of article *p*, we define their expertise *a*_*ip*_ using the top-*k* Medical Subject Heading (MeSH) terms from their previous publications. For each article *p*, we aggregated the collected list of MeSH terms (e.g., ‘B01.050.150.900.649’, ‘A08.186.211’, and ‘F03.615.400.100’), with each unique term individually denoted as *m*′. Additionally, the first-order primary subcategories referred to as ‘qualifiers,’ such as ‘B01’, ‘A08’, and ‘F03’ within the MeSH hierarchy, highlight the primary area of expertise. Let *M* denote the aggregated set of qualifiers associated with the article, *p*, and each qualifier be denoted as *m*_*i*_. Using these notations, Eq 3 provides the formulae for each attribute.

$$\begin{array}{cc} \text{Variety}\;\text{(N)} & :|\underset{m\in M}{⋃}m|\\ \text{Balance} & :\frac{-1}{\text{log}N}\times \sum_{m\in M}\text{Proportion}\left(m\right)\cdot \text{log}\;\text{Proportion}\left(m\right)\\ \text{Disparity} & :\frac{1}{c}\cdot \frac{1}{N^{2}}\sum_{k\neq l}d(m{\left(k\right)}^{\prime },m{\left(l\right)}^{\prime }) \end{array}$$ (3)

where,

$$\begin{array}{c} \text{Proportion}\left(m\right)=\frac{\text{count}(m,M)}{N}, \end{array}$$

$d(m_{k}^{\prime },m_{l}^{\prime })$ represents the edge distance between two MeSH terms $m_{k}^{\prime }$ and $m_{l}^{\prime }$, and *c* is a normalization factor, defined as the product of the maximum possible distance *d*_max_ between any two terms and the logarithm of the tree depth *h*, ensuring that the disparity measure is appropriately scaled to reflect the data’s complexity.

Finally, to compute expertise diversity, *d*_*exp*_, we utilize Eq 2, where *P*_*k*_ and *P*_*l*_ denote the proportion of paired MeSH qualifiers within the article’s aggregated list of MeSH qualifiers, *m* ∈ *M*, and *d*_*kl*_ denotes the edge distance between the MeSH terms, normalized by the same factor utilized in Eq 3, *c*. Lastly, for ethnic diversity, denoted as *d*_*eth*_, the Rao-Stirling index generalizes to the Gini-Simpson index (*G*) when any two entities are entirely dissimilar. This measure (*G*) is defined in Eq 4, where *q*_*i*_ signifies the proportion of each distinct diversity element, with *R* representing the aggregated count of unique elements.

$$\begin{array}{cc} & G=1-\Sigma_{1}^{R}q_{i}^{2} \end{array}$$ (4)

See Note 2.1 in S1 File for detailed descriptions and definitions of all diversity indices, including the decomposed attributes of expertise diversity. Note 2.2 in S1 File further explains their application in calculating the diversity indices.

## Supporting information

S1 File Supplementary file. This file includes the additional referenced tables and text definitions.
(PDF)

S1 Fig Mean scientific impact against diversity indices across journal types. Relationship between scientific impact, *RCR*, against all mean diversity indices. Each subplot includes individual data points based on a unique author count value and year of publication. Each regression has also been annotated with Pearson’s *r* and *p* values. This correlation is grouped by year and author count, and a correlation value of 0.48—0.7 suggests the presence of a quadratic relation.
(TIF)

S2 Fig Interaction between diversity indices and author count. Each curve indicates the varying estimates for diversity indices, *d*_*x*_:*x* ∈ (eth, gen, age, log. exp) for different values of the number of authors, indicating the presence of an interaction.
(TIF)

S3 Fig Model diagnostics. The plots indicate that residuals are approximately normal, with some deviations observed due to outliers. Deviance residuals are largely normal across fitted values, with a few high residuals.
(TIF)

S4 Fig Descriptive specification curve for each diversity index. Each subplot displays the ordered array of marginal estimates (including their interaction with author count) for the diversity indices across all specifications, with the horizontal line marking the estimate for the observed data. For diversity indices dependent on author count, *d*_*x*_ ∈ (eth, age, exp), the estimates total 81. In contrast, *d*_*x*_ = (gen), which is not dependent on author count, totals 27. The asterisk * indicates non-significant specifications.
(TIF)

S5 Fig Observed and expected specification curves for each diversity index. Each subplot displays the ordered estimates for the diversity indices across all specifications, comparing the observed and the expected under-the-null distribution. The expected curves are based on 50 shuffled samples where the key predictor, the diversity index value, is shuffled. All specifications are estimated on each shuffled sample, and the dashed lines depict the 2.5th, 50th, and 97.5th percentiles for each of these ordered estimates. The narrow confidence bands under the null for all diversity indices and the consistently low p-values indicate strong evidence for a robust and significant relationship for the observed data.
(TIF)
