# Supplementary material for: Patterns of diversity in biomedical coauthorships: An analysis across authors’ ethnicity, gender, age, and expertise
Source: PLoS One. 2025 Jan 31;20(1):e0316890. doi: 10.1371/journal.pone.0316890 (PMC11785319; doi:10.1371/journal.pone.0316890)
Supplement: S1 File — This file includes the additional referenced tables and text definitions. (PDF) [file pone.0316890.s001.pdf]

Supplementary Materials for
Patterns of diversity in biomedical coauthorships: An analysis across authors' ethnicity, gender, age, and expertise

Apratim Mishra [1], Haejin Lee[1], Sullam Jeoung[1], Vetle I. Torvik[1], Jana Diesner[1,2]
[1]School of Information Sciences, University of Illinois at Urbana-Champaign, Champaign, IL 61820.
[2]School of Social Sciences and Technology, Technical University of Munich, Munich, Germany.

This Supplementary file includes:

- Supplementary Tables - A, B, C, D, E, F, G, H, I, J
- Supplementary Text - 2.1, 2.2, 2.3, 2.4, 2.5

1 Supplementary Tables

Table A: Journal distribution in the main dataset.

Journal Name and Count					
Journal of Biological Chemistry	106390 (11.73%)	Proceedings of the National Academy of Sciences of the United States of America	63651 (7.02%)	Biochemical and Biophysical Research Communications	44099 (4.86%)
Biochemistry	36692 (4.05%)	Journal of Immunology	34391 (3.79%)	Biochimica et Biophysica Acta	34246 (3.78%)
Journal of Virology	28792 (3.17%)	Brain Research	27996 (3.09%)	Journal of Neuroscience	26172 (2.89%)
Nucleic Acids Research	23442 (2.58%)	Cancer Research	22930 (2.53%)	Journal of the American Chemical Society	22821 (2.52%)
Blood	22749 (2.51%)	Journal of Bacteriology	21992 (2.42%)	American Journal of Physiology	21211 (2.34%)
Annals of the New York Academy of Sciences	19465 (2.15%)	Methods in Molecular Biology	19211 (2.12%)	Journal of Urology	18777 (2.07%)
Annals of Thoracic Surgery	18154 (2.0%)	Journal of Molecular Biology	17876 (1.97%)	Infection and Immunity	17782 (1.96%)
Circulation	17231 (1.9%)	Biochemical Journal	16932 (1.87%)	Neurology	16026 (1.77%)
Cancer	15997 (1.76%)	American Journal of Cardiology	15518 (1.71%)	Molecular and Cellular Biology	15423 (1.7%)
Endocrinology	15070 (1.66%)	Analytical Chemistry	14270 (1.57%)	Science	14197 (1.57%)
Chest	13797 (1.52%)	Advances in Experimental Medicine and Biology	13731 (1.51%)	Journal of Pharmacology and Experimental Therapeutics	13398 (1.48%)
Journal of Applied Physiology	13029 (1.44%)	Nature	12967 (1.43%)	Pediatrics	12615 (1.39%)
American Journal of Obstetrics and Gynecology	11785 (1.3%)	The Lancet	11744 (1.29%)	The BMJ (British Medical Journal)	11010 (1.21%)
Physical Review Letters	3445 (0.38%)				

Table B: Comparing linear and quadratic fit for diversity indices against *RCR* in GLM regression model.

	Pseudo R Squared	
**Diversity indices**	**First Order**	**Second Order**
Ethnicity	0.005535	0.0063**
Gender	0.00033	0.00064**
Age	0.00055	0.00102**
Expertise	0.00242	0.0025**
** $p < 0.01$		
**The Likelihood Ratio Test Statistic(LRT) statistic has a **significant p-value for all cases.		

Table C: Fit statistics for the complete model after each step of adding a variable.

Variables	AIC	BIC	LLF	Df model	Df residual	Deviance	Pseudo R square
Diversity variables	3333011.861	-11233048.094	-1666496.931	8.000	907015.000	1209314.915	0.015
Journal impact	3060051.804	-11392050.714	-1530013.902	11.000	907012.000	1050271.141	0.145
Authors prior impact	3033364.699	-11405869.656	-1516669.349	12.000	907011.000	1036438.481	0.156
Journal impact	3015777.246	-11434916.016	-1507873.623	14.000	907009.000	1007364.686	0.180
Other Author Interaction	3008256.363	-11442590.755	-1504110.182	17.000	907006.000	999648.792	0.186
Country of article	3000194.067	-11447125.862	-1500069.034	27.000	906996.000	994976.506	0.190
Time lag	2993287.841	-11455798.425	-1496614.920	28.000	906995.000	986290.226	0.197
Time Novelty	2986567.931	-11458815.943	-1493252.965	30.000	906993.000	983245.271	0.199
Institution impact	2983729.575	-11460663.196	-1491831.787	32.000	906991.000	981370.582	0.201
Diversity Interaction	2982542.541	-11460957.416	-1491234.270	36.000	906987.000	981021.491	0.201
International	2982353.081	-11461030.128	-1491138.541	37.000	906986.000	980935.061	0.201
Author count	2982752.079	-11461926.613	-1491337.040	38.000	906985.000	980024.859	0.202
Funding	2983348.526	-11462628.478	-1491634.263	39.000	906984.000	979309.275	0.203
Abstract length	2981589.431	-11476152.420	-1495752.716	41.000	906982.000	965757.898	0.214

The model variables are added in a stepwise manner based on the lowest AIC for the newly added variable. The final model emerges as the optimal choice, demonstrating superior performance across multiple goodness-of-fit measures: lowest deviance (indicating better GLM fit), highest McFadden's pseudo R-squared (explaining more variance), and minimal BIC and AIC values (balancing model complexity and fit). While the penultimate model shows the lowest log-likelihood function (LLF), we adopt the final model due to its comprehensive optimization across multiple validation metrics and the large sample size.

Table D: Comparison of polynomial orders for different predictors

	R^2			AIC		
**Variable**	Order 1	Order 2	Order 3	Order 1	Order 2	Order 3
Time lag	0.0002	0.0002	0.0003	2909306.66	2909277.95	2909254.78
Author count	0.0284	0.0285	0.0286	2883382.06	2883258.74	2883227.26
Prior citation rate	0.0095	0.0099	0.0099	2900837.59	2900464.49	2900466.08
Abstract length	0.0359	0.0383	0.0390	2876376.20	2874067.44	2873455.76
Institution impact	0.0170	0.0192	0.0196	2893905.58	2891896.28	2891577.18
Paper Novelty	0.0041	0.0054	0.0055	2905803.80	2904557.49	2904508.73
Journal impact	0.0992	0.1059	0.1078	2814727.13	2808019.69	2806019.94

This table presents the comparative analysis of polynomial orders for each independent variable with the dependent variable, *RCR*. An increase in R squared (increase by 0.001) and a lower AIC determines the selection of the optimal order for each variable. Thus, Time lag, Author count, and Prior citation rate have first-order coefficients; Abstract length, Institution impact, and Paper Novelty have second-order; and Journal impact has third-order coefficients. Based on F-tests, all models are statistically significant at the $p < 0.05$ level.

Table E: Comparing correlation values for all variables in the dataset.

	Time lag	Author count	Prior citation rate	Abstract length	Institution impact	Paper Novelty	Journal Impact	Ethnic div.	Gender div.	Age div.	Log. Exper- tise div.	Variety	Balance	Disparity	International collab.	Funding
Time lag	1.00															
Author count	-0.18	1.00														
Prior citation rate	-0.12	0.12	1.00													
Abstract length	-0.20	0.18	0.09	1.00												
Institution impact	-0.04	0.14	0.17	0.04	1.00											
Paper Novelty	-0.23	-0.07	-0.01	-0.01	-0.03	1.00										
Journal Impact	-0.12	0.09	-0.01	-0.03	0.12	-0.04	1.00									
Ethnic div.	-0.07	0.16	0.08	0.09	0.22	-0.04	0.12	1.00								
Gender div	-0.10	0.25	-0.03	0.10	0.04	-0.06	0.01	0.14	1.00							
Age div.	0.00	0.22	-0.03	0.07	-0.01	-0.03	0.01	0.08	0.14	1.00						
Log. Expertise div.	-0.15	0.50	0.12	0.09	0.12	-0.18	0.15	0.18	0.18	0.05	1.00					
variety	-0.12	0.72	0.12	0.14	0.13	-0.08	0.02	0.14	0.20	0.07	0.28	1.00				
balance	0.15	-0.52	-0.28	-0.12	-0.12	0.03	-0.01	-0.09	-0.13	0.02	-0.56	-0.32	1.00			
disparity	-0.12	0.39	0.09	0.07	0.09	-0.15	0.17	0.15	0.14	0.04	0.86	0.06	-0.28	1.00		
International collab	-0.12	0.40	0.12	0.07	0.25	-0.01	0.08	0.13	0.10	0.02	0.28	0.34	-0.24	0.22	1.00	
Funding	0.07	0.00	0.11	0.05	0.26	-0.07	0.10	0.34	0.03	0.00	0.10	0.03	-0.00	0.09	0.04	1.00

Table F: Regression analysis of expertise diversity attributes - variety, balance, and disparity on scientific impact (RCR)

Variables	Simple Model [a]		Complete Model [o]	
	**Coef.**	**Std Err**	**Coef.**	**Std Err**
Constant			0.4906	0.006**
Time lag	0.035**	0.002	0.1294**	0.002
Author count	0.149**	0.002	0.0865**	0.006
Prior citation rate	0.122**	0.002	0.0941**	0.002
Abstract length	0.067**	0.002	0.1282**	0.002
Abstract length^2	-0.003*	0.001	-0.0143**	0.001
Institution impact	0.198**	0.002	0.0467**	0.002
Institution impact^2	-0.025**	0.001	-0.0030**	0.001
Paper Novelty	-0.022**	0.002	-0.0041*	0.002
Paper Novelty^2	0.059*	0.001	0.0397**	0.001
Journal impact	0.382**	0.002	0.3278**	0.002
Journal impact^2	0.082**	0.001	0.1086**	0.001
Journal impact^3	-0.002**	0.001	0.0216**	0.001
International	0.101**	0.002	0.0179**	0.003
Funding	0.125**	0.002	0.0690**	0.004
**Diversity indices**				
d_{eth}			-0.0137**	0.004
d_{eth}^2			-0.0044*	0.002
d_{gen}			0.0039**	0.004
d_{gen}^2			0.0198**	0.002
d_{age}			-0.0612**	0.004
d_{age}^2			-0.0095**	0.001
**Expertise Diversity**				
$variety$			-0.0264**	0.002
$variety^2$			0.0114**	0.001
$balance$			-0.0178**	0.002
$balance^2$			-0.0052**	0.001
$disparity$			-0.0586**	0.002
$disparity^2$			0.0088**	0.001
**Interaction terms**				
$d_{eth} \times d_{gen}$			-0.0168**	0.004
$d_{gen} \times d_{age}$			0.0117**	0.004
$d_{eth} \times$ Author count			0.0507**	0.005
$d_{age} \times$ Author count			0.0195**	0.006
**Fixed Effects**				
Country of publication			Included	
Journal type			Included	

Standard Error: ** $p < 0.01$; * $p < 0.05$; N = 907024

The regression table presents the relationship of the three attributes of d_{exp}: variety, balance, and disparity against scientific impact, RCR. [a] Simple model where only the singular independent variable is used for modeling (GLM with logarithm link). [o] Complete model includes all possible confounding variables.

Table G: Author ethnicities and their count in the dataset.

Ethnicity	Count	Ethnicity	Count
ENGLISH	393407 (24.4%)	DUTCH	32541 (2.02%)
OTHER	193166 (11.98%)	KOREAN	32489 (2.02%)
CHINESE	185379 (11.5%)	ARAB	31809 (1.97%)
JAPANESE	138803 (8.61%)	ISRAELI	14229 (0.88%)
GERMAN	132349 (8.21%)	ENGLISH-GERMAN	12701 (0.79%)
HISPANIC	90286 (5.6%)	GERMAN-ENGLISH	11574 (0.72%)
FRENCH	89404 (5.55%)	GREEK	10314 (0.64%)
ITALIAN	68365 (4.24%)	ENGLISH-FRENCH	10043 (0.62%)
INDIAN	55123 (3.42%)	TURKISH	8348 (0.52%)
SLAV	51483 (3.19%)	ENGLISH-CHINESE	7455 (0.46%)
NORDIC	42850 (2.66%)		

The top 20 ethnicities are collected. For computing the diversity measure when using paired ethnicities (e.g., English-Italian), they are considered equally important .

Table H: Author ethnicities and their gender distribution.

Ethnicity	Unknown	F	M
ENGLISH	6.83%	35.40%	57.77%
OTHER	16.16%	37.29%	46.55%
CHINESE	70.36%	9.38%	20.26%
JAPANESE	12.87%	18.41%	68.73%
GERMAN	6.22%	31.57%	62.21%
HISPANIC	7.67%	42.46%	49.88%
FRENCH	5.78%	40.33%	53.89%
ITALIAN	4.87%	44.45%	50.68%
INDIAN	26.16%	27.01%	46.83%
SLAV	10.08%	40.51%	49.40%
NORDIC	5.91%	39.23%	54.85%
KOREAN	55.59%	16.13%	28.29%
DUTCH	11.13%	31.22%	57.65%
ARAB	15.79%	23.68%	60.52%
ISRAELI	11.24%	34.04%	54.72%
ENGLISH-GERMAN	5.02%	29.11%	65.87%
GERMAN-ENGLISH	7.36%	36.64%	56.00%
GREEK	7.38%	32.06%	60.56%
ENGLISH-FRENCH	8.65%	39.16%	52.19%
TURKISH	15.26%	27.17%	57.57%
ENGLISH-CHINESE	7.07%	37.04%	55.89%

Certain ethnicities (primarily Asian) result in a high percentage of gender predicted 'Unknown.'

Table I: Country of affiliation for all papers.

Country Name and Count			
USA	375246 (41.37%)	Sweden	11899 (1.31%)
Japan	76368 (8.42%)	Belgium	11089 (1.22%)
Germany	58375 (6.44%)	Switzerland	10819 (1.19%)
Canada	53945 (5.95%)	Israel	10620 (1.17%)
UK	50969 (5.62%)	Denmark	9167 (1.01%)
France	45784 (5.05%)	India	7603 (0.84%)
OTHER	34141 (3.76%)	Austria	7551 (0.83%)
Italy	26349 (2.9%)	Taiwan	6528 (0.72%)
Australia	23422 (2.58%)	Finland	6146 (0.68%)
China	22340 (2.46%)	Brazil	5892 (0.65%)
Netherlands	17853 (1.97%)	Argentina	3139 (0.35%)
Spain	13654 (1.51%)	Norway	3030 (0.33%)
Korea	12387 (1.37%)	Poland	2708 (0.3%)

Table J: Overview of original and alternative specifications used in the analysis.

No.	Decision	Original Specification	Alternative Specifications
1	Measure of scientific impact	Relative Citation Ratio	Alternative measure: Citation rate
2	Operationalization of ethnicity and gender	Ethnea and Genni	Alternative measure: Ethniccolr and gender-guessor
3	Operationalization of expertise	Rao-Stirling index	DIV index as an alternative measure
4	Control variables	All relevant variables included in the model	Exclusion of individual variables iteratively; only first-order terms considered
5	Data subsets	Full dataset	Data subsets when all authors have predicted ethnicity only, predicted gender only, and both predicted ethnicity and gender
6	Type of regression model	Tweedie regression model	Gamma regression model; Tweedie with inverse log link; Tweedie model with variance powers of 1.2, 1.5, and 1.8

2 Supplementary Notes

2.1 Data

The data used in this analysis is based on a snapshot of PubMed in 2018, labeled 'Authority 2018' [1]. 'Authority 2018' includes a total of 29.1 million article records and 114.2 million author name instances. Features of this snapshot include: unique author ID (referred to as auid), last name (variants), first name (variants), email addresses, ORCIDs, top 20 affiliation words, top 20 most frequent MeSH, journal names with count, co-author names, h-index, citation counts, among others. In this snapshot, each instance uniquely represents an author (e.g., 10888_3 represents an author where 10888 refers to a unique paper and 3 is the author's positional information on that paper). To conduct our analysis, we initially picked a random assortment of authors and then sampled a list of papers. We then further narrowed down the papers (represented by a unique pmid) by focusing on 1991-2014 and selecting the foremost 40 journals available in the snapshot. We classify journals into medicine, science, and biology, as illustrated in the study [2]. Additionally, as before 2014, PubMed only offered author affiliation for the first author (PubMed update).

2.1.1 Classifying the ethnicity of an author

Our research uses "Ethnea," an instance-based ethnicity classifier, to identify author ethnicities [3]. This method uses a nearest-neighbor algorithm to identify the author's ethnicity (or a pair of ethnicities) and gives a probabilistic alignment to a total set of 26 predefined ethnicities. Unlike machine learning-based prediction methods, "Ethnea" is based on a lookup approach where authors' names are geo-coded and associated with countries globally. This approach leans towards nationality identification and captures dual ethnicities, often resulting from marriage or migration. Therefore, it can make accurate predictions for names that are rare in the US but common in other countries. The large availability of data across over 200 countries and over 20 years improves its reliability compared to feature-based methods. In cases where authors are assigned dual ethnicities, such as "ENGLISH-FRANCE," both ethnicities have equal weight. Hence, the author's ethnic background is represented as if there were two authors, one with the ethnicity "ENGLISH" and the other "FRANCE." Our analysis only considers the top 20 ethnicities; the rest are pooled as 'OTHER' (See Supplementary Table G for ethnicity count). Additionally, Supplementary Table H gives the gender distribution for each category, depicting the relationship between ethnicity and gender prediction through the variability in gender prediction due to author nationality. Authors from Asian countries (Chinese, Japanese, and Korean) have a higher prediction of 'unknown/unisex' depicting the complexities in gender prediction from English names.

We also utilize the repository 'ethnicolr,' an alternative ethnicity prediction method, as part of the specification analysis [4]. Specifically, we use the Wikipedia data-based machine learning model for ethnicity prediction, which uses the full name to predict ethnicities, with the data scraped from a database of 140,000 name/race associations [5]. This method gives probabilistic mapping to one of 13 categories, which differs from our primary method of ethnicity prediction, 'Ethnea.' The unique predicted categories are: "Asian, GreaterEastAsian, EastAsian," "Asian, GreaterEastAsian, Japanese," "Asian, Indian subcontinent," "GreaterAfrican, Africans," "GreaterAfrican, Muslim," "GreaterEuropean, British," "GreaterEuropean, EastEuropean," "GreaterEuropean, Jewish," "GreaterEuropean, WestEuropean, French," "GreaterEuropean, WestEuropean, Germanic," "GreaterEuropean, WestEuropean, Hispanic", "GreaterEuropean, WestEuropean, Italian", "GreaterEuropean, WestEuropean, Nordic."

2.1.2 Classifying the gender of an author

We utilize "Genni" to identify the gender of authors [3]. "Genni" measures the gender orientation based on the first name over time on a set of search engine queries (such as "Uncle Taylor") with the name prefixed by masculine and feminine markers (e.g., "Mother Jean") and on the U.S. SSA (Social Security Administration). The possible gender classifications are "Male," "Female," and "Unknown." In the SSA (U.S Social Security Administration) dataset, "Genni" misclassifies 2.25% of the unique people (compared to the 1.74% error rate) but is also applicable for names outside the United States SSA. For certain international names, Genni performs better than the SSA data set as it is based on web searches that apply to the broader worldwide web. The misclassification rate has been higher in recent years due to the growing diversity in names for typical English names (for example, Will), names from specific countries (Asian countries), and atypical names. However, it is important to note that the model results in "unknown/unisex" to avoid false positives. Additionally, it makes use of "Ethnea" to determine the

gender for some names that are unisex but gender-specific due to the location of the person (for example, English *Andrea's* are labeled female, and Italian *Andrea's* are labeled Male).

The alternative method to predict gender in our specification analysis is 'gender-guessor,' gender-guesser) which is a Python-based library based on a wrapper around a fixed set of 45,000 names [6]. The distinct categories are unknown, andy (androgynous), male, mostly_male, female, and mostle_female. The 'unknown' prediction corresponds to names that are not available in the library repository. Additionally, it offers parameters for 'location_id' to present a more suitable prediction. When considering names with a predicted gender, 'Genni' aligns with 'gender-guessor' for 94.4% of authors. Compared to gender inference services available for a subscription, 'gender-guessor' performs better on metrics ignoring 'unknowns,' achieving 2.6% misclassification on observed data [7].

2.1.3 Classifying academic age of an author

The "age" of an author is specific to each article and is equal to the number of published papers from the time of an author's first article to a specific subsequent article p. Consequently, an author's "age " varies for each publication. The age is then divided into bins to be able to use the Rao-Stirling index to calculate the age diversity index:

1. Age group 1: 0 years
2. Age group 2: 1 - 3 years
3. Age group 3: 4 - 10 years
4. Age group 4: 11 - 20 years
5. Age group 5: 21 - 50 years
6. Age group 6: 51 - 100 years
7. Age group 7: 101 - 200 years
8. Age group 7: >=201 years

Academic age in the binned form presents a rationale for an index emphasizing the ordinal order of the age (the age gap between bin one and bin two is very different than the gap between bin one and bin seven).

2.1.4 Classifying expertise of an author

In PubMed, MeSH terms are part of an organized, controlled vocabulary defined by experts to label individual papers. PubMed serves as a human-curated database where an individual indexes each article, and the MeSH terms used to define an article are an effective method for seeking semantic context. The terms are practical as they search for meaning rather than the mere occurrence of a specific word. Additionally, they are pre-defined from a database and include synonyms, making them pragmatic and comprehensive. They establish concepts and contain alternative spellings, and every article (identified by a unique pmid) is distinguished by a set of terms that may be related as parent or child terms in a hierarchical tree structure. E.g., the MeSH term *Lung Neoplasms* includes all related variations, such as lung cancer, lung tumor, lung neoplasm, and pulmonary cancer.

Every time an author publishes a paper, the author gains knowledge, symbolized by the MeSH terms associated with a specific article. As a result, every author has an "expertise" for a specific set of MeSH terms. Next, we choose the top n MeSH terms (in our case, n = 12) by frequency for an author to represent the knowledge concepts over which the author has significant "expertise." Consequently, an author is associated with expertise in a set of MeSH terms for each article. The expertise attributed to an author may vary with each publication they produce, based on the frequency of unique MeSH terms. For instance, for a particular article (pmid), the first author might have expertise linked with the MeSH terms such as 'Humans,' 'Brain', ..., 'Amyloid,' and 'Alzheimer Disease.' Correspondingly, these MeSH terms align with values from the MeSH tree, such as 'B01.050.150.900.649.313.988.400.112.400.400', 'A08.186.211', 'F03.615.400.100', and 'B01.050', and would form the expertise of the author.

For a particular article, p, let all authors be represented as $Authors(p)$. Now, for any given author, $a_i \in Authors(p)$ in that article, let $mesh(a_{ip})$ denote the aggregated set of MeSH terms for that particular

author up to a certain article, p. Therefore, for the article, p, "expertise" is represented as a list of lists and can be represented as:

$$\begin{aligned} &expertise(p) = [\text{mesh}(a_{ip}) : a_i \in \text{Authors}(p)] \\ \text{where } &mesh(a_{ip}) = \{\text{MeSH terms for author } a_i \text{ up to the article } p\} \end{aligned} \quad (1)$$

2.1.5 Classifying expertise diversity: Variety

"Variety" refers to the number of unique categories into which the system elements are divided. In our study, variety for an article refers to the number of unique MeSH qualifiers in a paper. In our study, for a particular author, a_i, let $mesh(a_{ip})$ denote the list of aggregated MeSH terms (top k=12) for a particular article, p. To calculate variety, we consider the immediate MeSH qualifier of the terms as distinct values. For example, given the MeSH terms 'B01.050.150.900.649.313.988.400.112.400.400', 'A08.186.211', 'F03.615.400.100', the distinct MeSH qualifiers would 'B01', 'A08', and 'F03'. We use the term $meshsub(a_{ip})$ to symbolize the set of MeSH qualifiers for an author, a_i, associated with an article, p. Then, variety is given by:

$$\begin{aligned} &\text{variety}(p) = \left| \bigcup_{a_i \in Authors(p)} meshsub(a_{ip}) \right| \\ &\text{where, } meshsub(a_{ip}) \text{ is set of MeSH qualifiers for author, } a_i, \text{ for article, } p \\ &\text{and, variety is the cardinality of the set, also now denoted as } N. \end{aligned} \quad (2)$$

This computation of variety aligns with the variety measure presented in the main submission (Equation 3), where variety represents the total number of unique MeSH qualifiers associated with each article. Specifically, the variety calculated above also equals $\left| \bigcup_{m \in M} m \right|$ in equation 3, where m denote each MeSH qualifier inherently related to the article p derived from the set M.

2.1.6 Classifying expertise diversity: Balance

"Balance" refers to the distribution of unique categories for the publication. For an article, p, the set of MeSH qualifiers for any author, a_{ip}, is given by $meshsub(a_{ip})$. Aggregating across all authors, let M be the concatenation of all author MeSH qualifiers, $M = \bigcup_{a_i \in Authors(p)} meshsub(a_{ip})$. Let m denote each MeSH qualifier inherently related to the article p derived from the set M, and let $P_j(m)$ indicate the proportion of each m. Then balance can be denoted by:

$$\begin{aligned} \text{balance}(p) &= -\frac{1}{\log N} \sum_{m \in M} P_j(m) \log P_j(m) \\ \text{where } P_j(m) &= \frac{\text{count}(m, M)}{N} \\ \text{and } M &= \bigcup_{a_i \in Authors(p)} meshsub(a_{ip}) \end{aligned} \quad (3)$$

2.1.7 Classifying expertise diversity: Disparity

"Disparity" refers to the degree to which individual categories differ based on specific traits or attributes. In our study, to compute a distance measure between pairs of MeSH terms, we view the lowest identifiable part of the MeSH term as the considered attribute. The distance between two nodes is defined by the number of edges traversed to get from one node to the other. For example, the edge distance between the MeSH terms 'B01.150.900.649.313' and 'B01.150.400' is 4. Moreover, there are 16 main heading terms (called descriptors) in MeSH; any two headings, such as Organisms[B] and Health Care[N], are at a unit distance (disparity) from each other.

Let $mesh(a_{ip})$ denote the aggregated set of MeSH terms for any specific author up to a certain article, p, and N denote the variety (cardinality of MeSH qualifiers). Aggregating across all authors, we get $M^{'}$, which is the concatenation of all author MeSH terms, $M^{'} = \bigcup_{a_i \in Authors(p)} mesh(a_{ip})$. Let $m^{'}$ be a unique MeSH term inherently related to the article p, belonging to the set $M^{'}$. If d is the edge distance between any 2 MeSH terms, $m(k)^{'}$ and $m(l)^{'}$, and c is the normalization constant, defined as the maximum possible distance (22) multiplied by the logarithm of the tree depth, to ensure that the disparity measure is scaled appropriately given the complexity of the data, then disparity can be denoted by:

$$\text{disparity}(p) = \frac{1}{c}.\frac{1}{N^2}\sum_{k \neq l} d(m(k)^{'}, m(l)^{'}) \tag{4}$$

2.2 Quantifying Diversity value

This section discusses the two primary diversity measures (along with an additional measure) that are considered in our study to calculate the four diversity indices: gender (d_{gen}), ethnicity (d_{eth}), age (d_{age}), and expertise (d_{exp}).

2.2.1 Rao's quadratic entropy

The Rao-Stirling index (denoted as Q) is one of the most widely applied diversity measures to measure functional diversity. It is calculated by summing up the pairwise distance between the two categories (for the case, d_{exp} the distance is between pairwise MeSH terms), i, and j (represented as d_{ij}). This distance is weighted by the relative abundance of both categories (P_i and P_j). Let S denote the complete set of authors for all articles under consideration P. Furthermore, let $Authors(p)$ denote the set of authors for an article p. For any given article, p, any set of authors for an article $x(s_i) : s_i \in Authors(p)$ such as that $x \in \{\text{exp, gen, age}\}$ is a multiset of values. For example, for any given article p, with n total authors, we have:

$gen(s_i) : s_i \in Authors(p) =$ [M, M, F, U].
$age(s_i) : s_i \in Authors(p) =$ [2, 3, 1, 6].
$exp(s_i) : s_i \in Authors(p) =$ [$mesh(a_{1p})$, $mesh(a_{2p})$,..., $mesh(a_{np})$](from eq. 1)

We define gender diversity among authors, d_{gen}, defining that 'Male' and Female' are completely distinct (distance is 1) and 'unknown' might be non-binary or not specified, making it somewhat similar to both 'Male' and 'Female' (distance of 0.5). For d_{age}, we use binned values to group the academic age of an author, and the distance term denotes the absolute difference between bins. For example, for the case of gender, for any multiset, M, let the notation $|M|$ represent the cardinality of M. The term $set(M)$ denotes the foundational set of M, and let $set(m, M)$ denote the frequency of element m in M. Therefore, if given M = [Male, Male, Female, Unknown], we have $|M| = 4$, set(M) = [Male, Female, Unknown], set(Male, M) = 2, set(Female, M) = 1 and set(Unknown, M) = 1. In this case, the Rao-Stirling index of a multiset, M would equate to:

$$\begin{aligned} &\text{Rao(M)} = \sum_{m1, m2 \in set(\text{M})} \text{proportion}(m1, M) \times \text{proportion}(m2, M) \times d_{m1,m2} \\ &\text{where , proportion}(m, M) = \frac{set(m, M)}{|M|} \end{aligned} \tag{5}$$

Here, d_{ij} is the distance between the unique elements of M, m1 and m2. For the case of d_{exp}, using equation 1, we aggregate the list of all MeSH terms across all authors. This can be represented by:

$$expertise(p) = [\text{mesh}(a_{ip}) : a_i \in \text{Authors}(p)] \tag{6}$$

Here, we concatenate the list of individual author-based MeSH terms, effectively returning a single set of MeSH terms across all authors. Now, we can use the Rao-Stirling index to compute the expertise diversity for any article p, given by:

$$Q(p) = \sum_{k \neq l} d(m(k)^{'}, m(l)^{'}).P_k(m)P_l(m) \tag{7}$$

For an article, p, P_k and P_l is the proportion of each pairwise unique MeSH qualifier within the article's aggregated list of MeSH qualifiers, $m \in M$, as represented in equation 3. Here, d_{kl} denotes the edge distance between any two MeSH terms from the aggregated list of MeSH terms, $m^{'} \in M^{'}$, as represented in equation 4. This result is then normalized by a constant c, set to 22 based on the maximum distal value, as demonstrated in equation 4. Using this notation, we can now compute the diversity indices, $d_x \in$ (exp, age, gen) for a publication.

2.2.2 Gini-Simpson Index

We use the Gini-Simpson index to measure the ethnicity diversity index. This index is a quantitative measure of diversity that reflects how many different types (such as ethnicity: English, FRENCH, INDIAN) are in the data set (each unique paper). Given the d_{ij} between the pair of unique categories is 1, Rao's Q and the Gini-Simpson Index are mathematically equivalent. If an ethnicity contains a hyphen, it is split into two distinct ethnicities. Using the same notation as in section 2.2.1,for any given article p, we have:

$$eth(s_i) : s_i \in Authors(p) \rightarrow [\text{split}(e) | e \in Ethnicities(s_i)].$$

Here, split(e) refers to a function that takes an ethnicity e and returns a single ethnicity e if there is no hyphen and two separate ethnicities if e contains a hyphen.

For any multiset, M, let the notation $|M|$ represent the total number of elements in M and let $set(m, M)$ denote the frequency of element m in M. Given a multiset M = [English, Chinese, English, German], we have $|M| = 4$, set(M) = [English, Chinese, German], set(English, M) = 2 and set(Chinese, M) = 1. Here, the Gini-Simpson index of a multiset, M would equate to:

$$\begin{aligned} Gini(\text{M}) = & \quad 1 - \sum_{m \in set(\text{M})} proportion(m, M)^2, \text{ where} \\ proportion(m, M) = & \quad \frac{set(m, M)}{|M|} \end{aligned} \tag{8}$$

This notation can compute a particular article's diversity index, $d_x \in (eth)$.

2.2.3 DIV Index

We utilize the *DIV* index as an alternative measure of diversity, which computes variety, balance, and disparity independently and then combines them *ex post* [8, 9]. The variety dimension captures the number of distinct categories (such as expertise domains), balance is operationalized through the Gini coefficient to measure the evenness of distribution across these categories, and disparity reflects the conceptual differences between categories. By integrating the three dimensions separately, the *DIV* index provides a more nuanced understanding of diversity, addressing limitations found in earlier approaches such as the Rao-Stirling index, which preemptively combines variety and balance into a single measure, often leading to interpretive ambiguities. The *DIV* index also adheres to the monotonicity principle, whereby an increase in one component (e.g., balance) results in a proportional increase in overall diversity, provided the other components remain constant [10]. This avoids issues encountered in earlier indices, such as the failure of the Rao-Stirling index to account for balance fully. By independently operationalizing balance, variety, and disparity, this method offers a more straightforward and robust measure of diversity, making it better suited for assessing interdisciplinarity and knowledge integration. In our study, the *DIV* index captures expertise diversity within co-authorship networks, offering a refined and improved perspective on how varied expertise across co-authors can contribute to scientific impact. Equation 9 represents the alternative index for any article, p:

$$DIV = \text{variety}(p) * \text{balance}(p) * \text{disparity}(p) \tag{9}$$

2.3 Randomized Model to examing homophily and heterophily

To study homophily for the four diversity indices, $d_x \in$ (eth, gen, age, exp), we attempt to isolate the effects of the confounding factor while preserving the characteristics of other variables. To add some additional notation, for every article, $p_i \in P$, let $year(p_i)$ refer to the year of publication, and let n refer to the number of authors for the paper. Next, let us denote by $S_{n,y} \subset S$, the set of all authors for an article with n authors published in year y. Therefore,

$$S_{n,y} = \bigcup_{p_i \in P; Authors(p_i)=n, Year(p_i)=y} \{s \in Authors(p_i)\} \tag{10}$$

With the specific set of authors, $S_{n,y} \subset S$, we can randomize this set to check trends for homophily and heterophily. We will now discuss the randomization process for ethnic diversity, d_{eth}; the other diversity indices can also follow the same process. Let $eth(a)$ denote the ethnicity of an author a. Then, for each shuffled step with the number of authors, n, and publication year, y, the ethnicities in $S_{n,y}$ are shuffled as follows:

- Create a list of all authors, $L_{n,y}$, where the $L_{n,y}[i] \coloneqq eth(a_i), \forall a_i \in S_{n,y}$
- Create a shuffled list , $L^{'}_{n,y}$
- Set ethnicities for the new shuffled list of authors as $eth(a_i) \coloneqq L^{'}_{n,y}[i], \forall a_i \in S_{n,y}$

To display the distribution of the observed ethnicity index, values are binned across possible diversity values. For each shuffled set, the randomized set of computed diversity index is allotted to the nearest bin. This randomization process is repeated 100 times for each diversity index, and a mean value for each bin gives us the overall distribution of the randomized diversity index. In the randomization process, we do not average the computed randomized diversity index for individual articles across the 100-time steps. For example, each article p does not have an analogous randomized article $p\prime$ for this process; however, the distribution of the entire data is a randomized set. Our method ensures the diversity index spans the entire range of possible values of diversity 0 - 1. Finally, based on the bin distributions, the randomized data set for $d_x \in$ (eth, gen, age, exp) can be compared with the observed real value.

2.4 Institution characteristics

In this study, we analyze two distinct institution characteristics (impact and funding) and their relationship with diversity indices, $d_x \in$ (eth, gen, age, Log. exp). Specifically, we compute the publication rate (citations per year) for each unique institution in the dataset at a given time (2023). Due to the disambiguation process used for institutions, which relies on a unique grid ID based on geolocation, we cannot directly link institution names to standardized academic rankings, such as the Scimago or Shanghai rankings. These rankings assess institutions on multiple criteria, including research quality, citation rates, journal impact, and international collaboration. Thus, the variable 'Institution impact' in our regression analysis represents the highest publication rate among all authors' institutions associated with a given paper.

Since paper-specific funding data is unavailable, we utilize a disambiguated funding dataset for authors, indicating funding status for researchers in a specific year. Accordingly, the variable 'Funding' in the regression model is a binary indicator of whether any author of a specific article are funded in that year (not necessarily for the given paper).

2.5 Specification Analysis

Given that statistical tests often rely on analytical choices that may be seen as arbitrary or biased, we have carefully outlined specific model specifications to ensure the validity and robustness of our findings. By employing a specification curve analysis, we assess how our results hold across a range of reasonable model specifications to validate the conclusions of our study [11]. Our study utilizes a specification set of 81 variations, encompassing the following elements: (a) Outcome variables, including different measures of scientific impact (*RCR* and citation rate); (b) Predictive algorithms for estimating ethnicity and gender [12, 13]; (c) Varying operationalization of the diversity measure used for computations (such as Rao-Stirling index and the DIV indicator) [14]); (d) Control variables, with different sets of covariates

and using data subsets (dropping each covariate, and dropping cases with 'unknown' gender and ethnicity prediction); and (e) Statistical models, such as Tweedie and Gamma regression with different link functions (log and inverse-log) [15] (Table J in S1 File includes details on all specifications). As this study focuses on exploring how diversity indices among authors ($d_x \in$ eth, gen, age, exp) are associated with scientific impact, S4 Fig illustrates the marginal effect for each diversity index across all specifications (assuming, no interaction between any pair of diversity indices).

To test the robustness of the relationship between diversity in research co-authorships and scientific impact, we conducted a specification curve analysis with 81 variations, examining the estimates of four diversity indices: ethnicity, gender, academic age, and expertise. For ethnicity, academic age, and expertise, 81 specifications were analyzed, reflecting their interaction with the author count. In contrast, for gender, only 27 specifications were considered, as its estimate is independent of author count. Firstly, our analysis highlights a significant interaction between author count and the diversity indices, revealing the necessity of including author count as a covariate to prevent biased outcomes (author count at different standard deviations (SD): -1, 0, and +1). Importantly, the observed model's estimates for all diversity indices align closely with the median values across the specifications, suggesting that all our estimates are robust and stable. Ethnic diversity loses significance when 1) citation rate is the dependent variable coupled with the alternative measure of expertise diversity (DIV), and 2) the dataset is sampled based on all predicted values of researchers' gender and ethnicity, remaining significant in 75 out of 81 cases. In the latter case, the p-value of 0.07 does suggest a marginal effect, approaching significance but not meeting the conventional threshold ($p < 0.05$). This borderline result indicates a potential underlying relationship, but with some uncertainty, and highlights the sensitivity of sampling choices.

Additionally, excluding the year of publication as a covariate leads to an outlier estimate, suggesting that the year of publication is a significant variable in modeling impact, especially at higher levels of author interaction. The gender diversity index shows consistent and stable results. However, small outliers are observed when 1) only predicted values (M or F) for gender make the sample, and 2) only predicted values (M or F) for gender and ethnicity (no 'Other" category) make the sample. Despite these outliers, the relationship remains significant, stable, and positive for most specifications (25 out of 27), highlighting the influence of gender-diverse teams on scientific outcomes. The age diversity index similarly exhibits a robust relationship with scientific impact, with no sign changes and statistically significant results across all specifications. However, an outlier appears when author count is excluded as a covariate, though it maintains the same sign, suggesting a stable but sensitive relationship. Expertise diversity shows some sensitivity to model specifications, with sign changes occurring when 1) citation rate is analyzed alongside the DIV indicator (an alternative measure of expertise diversity) across all three author interaction standard deviation (SD) values, and 2) the DIV indicator (expertise diversity measure) alone is used, resulting in sign changes in two out of three SD values. Additional sign changes appear at high author interaction SD values when 3) different predicted algorithms are used for ethnicity and gender author classiciation. Despite this variability, 68 out of 81 specifications retain the same sign and remain statistically significant, suggesting that expertise diversity's association with scientific outcomes is mostly stable but can vary with certain operationalizations of expertise, ethnicity, and gender.

Next, the robustness is further assessed using three key statistics: (1) the median effect size across all specifications, (2) the share of significant results, and (3) the aggregation of all P-values through Stouffer's method. To generate null distributions, we shuffle the diversity index multiple times to generate it under the null dataset. Our findings consistently indicate that gender and expertise diversity positively influence scientific impact, while ethnicity and age diversity negatively correlate with impact. See S5 Fig for details on each diversity index's observed and under-the-null curve. The comparison with the null distributions further highlights the validity of these results and reinforces the robustness of the observed estimates, which cannot be observed due to random chance.

References

[1] Torvik V, Smalheiser N. Author-ity 2018 - PubMed author name disambiguated dataset; 2021. Available from: https://doi.org/10.13012/B2IDB-2273402_V1.

[2] Mishra S, Fegley BD, Diesner J, Torvik VI. Self-citation is the hallmark of productive authors, of any gender. PLOS ONE. 2018;13(9):1–21. doi:10.1371/journal.pone.0195773.

[3] Smith BN, Singh M, Torvik VI. A search engine approach to estimating temporal changes in gender

orientation of first names. In: Proceedings of the 13th ACM/IEEE-CS joint conference on Digital libraries; 2013. p. 199–208.

[4] Laohaprapanon S, Sood G, Naji B. ethnicolr: Predict Race and Ethnicity From Name; 2022. Available from: https://github.com/appeler/ethnicolr.

[5] Ambekar A, Ward C, Mohammed J, Male S, Skiena S. Name-ethnicity classification from open sources. In: Proceedings of the 15th ACM SIGKDD international conference on Knowledge Discovery and Data Mining; 2009. p. 49–58.

[6] Michael J. 40000 namen, anredebestimmung anhand des vornamens. C't. 2007; p. 182–183.

[7] Santamaría L, Mihaljević H. Comparison and benchmark of name-to-gender inference services. PeerJ Computer Science. 2018;4:e156.

[8] Leydesdorff L, Wagner CS, Bornmann L. Interdisciplinarity as diversity in citation patterns among journals: Rao-Stirling diversity, relative variety, and the Gini coefficient. Journal of informetrics. 2019;13(1):255–269.

[9] Leydesdorff L, Ivanova I. The measurement of "interdisciplinarity" and "synergy" in scientific and extra-scientific collaborations. Journal of the Association for Information Science and Technology. 2021;72(4):387–402.

[10] Rousseau R. The repeat rate: from Hirschman to Stirling. Scientometrics. 2018;116(1):645–653.

[11] Simonsohn U, Simmons JP, Nelson LD. Specification curve analysis; 2020.

[12] Laohaprapanon S, Sood G, Naji B. ethnicolr: Predict Race and Ethnicity From Name; 2022. Available from: https://github.com/appeler/ethnicolr.

[13] Michael J. 40000 namen, anredebestimmung anhand des vornamens. C't. 2007; p. 182–183.

[14] Leydesdorff L, Wagner CS, Bornmann L. Interdisciplinarity as diversity in citation patterns among journals: Rao-Stirling diversity, relative variety, and the Gini coefficient. Journal of informetrics. 2019;13(1):255–269.

[15] McCullagh P. Generalized linear models. Routledge; 2019.
