# Supplementary figures and images for: Patterns of diversity in biomedical coauthorships: An analysis across authors’ ethnicity, gender, age, and expertise
Source: PLoS One. 2025 Jan 31;20(1):e0316890. doi: 10.1371/journal.pone.0316890 (PMC11785319; doi:10.1371/journal.pone.0316890)

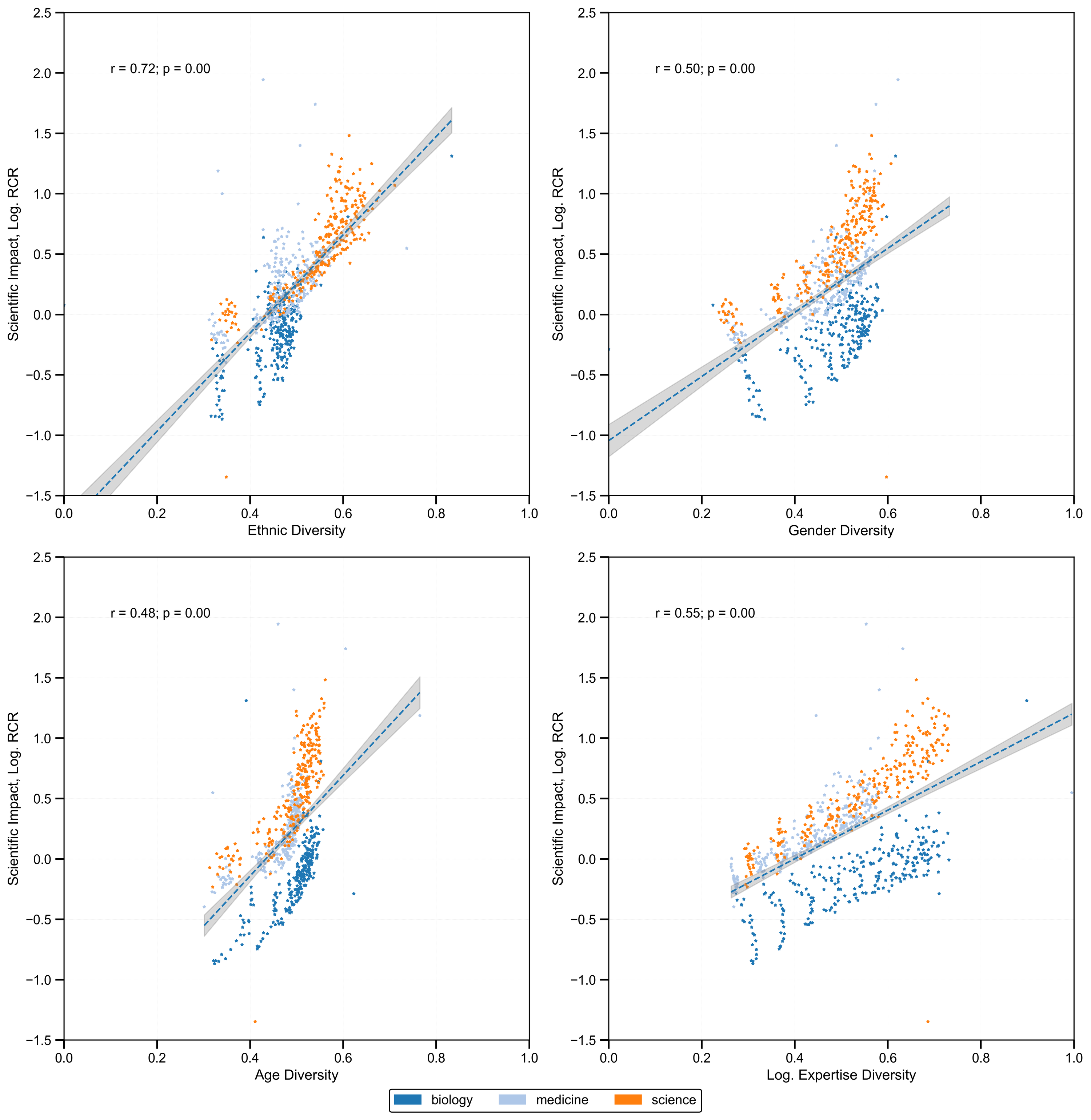

Supplement: S1 Fig — Relationship between scientific impact, RCR, against all mean diversity indices. Each subplot includes individual data points based on a unique author count value and year of publication. Each regression has also been annotated with Pearson’s r and p values. This correlation is grouped by year and author count, and a correlation value of 0.48—0.7 suggests the presence of a quadratic relation. (TIF) [file pone.0316890.s002.tif]

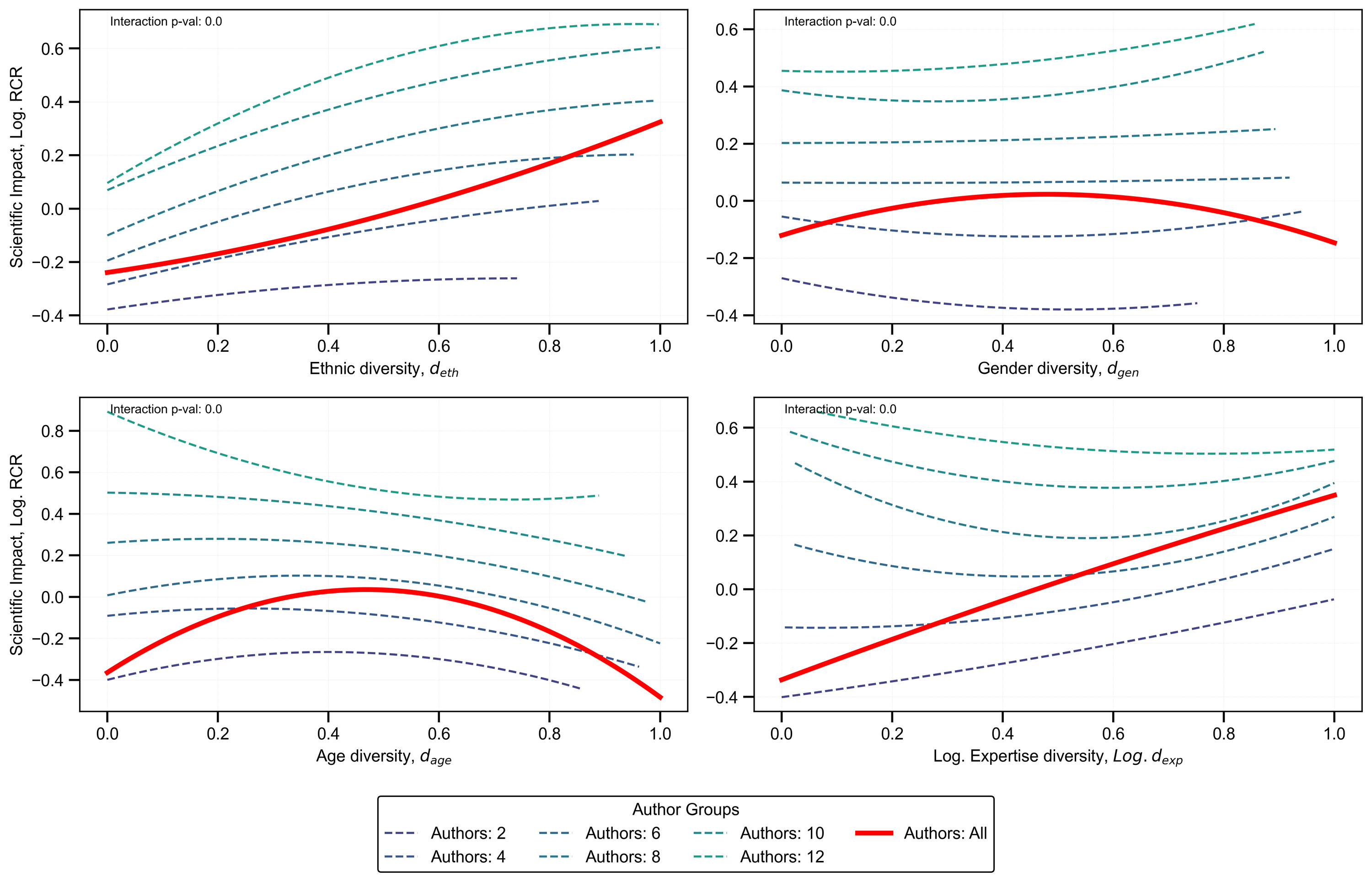

Supplement: S2 Fig — Each curve indicates the varying estimates for diversity indices, dx:x ∈ (eth, gen, age, log. exp) for different values of the number of authors, indicating the presence of an interaction. (TIF) [file pone.0316890.s003.tif]

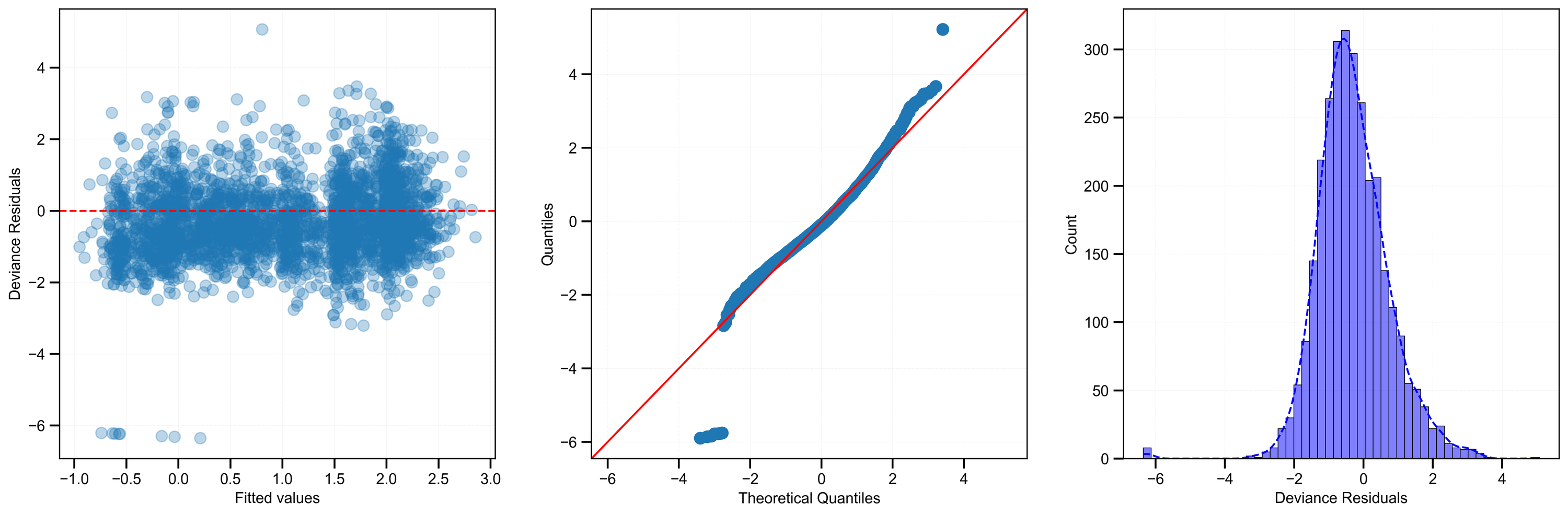

Supplement: S3 Fig — The plots indicate that residuals are approximately normal, with some deviations observed due to outliers. Deviance residuals are largely normal across fitted values, with a few high residuals. (TIF) [file pone.0316890.s004.tif]

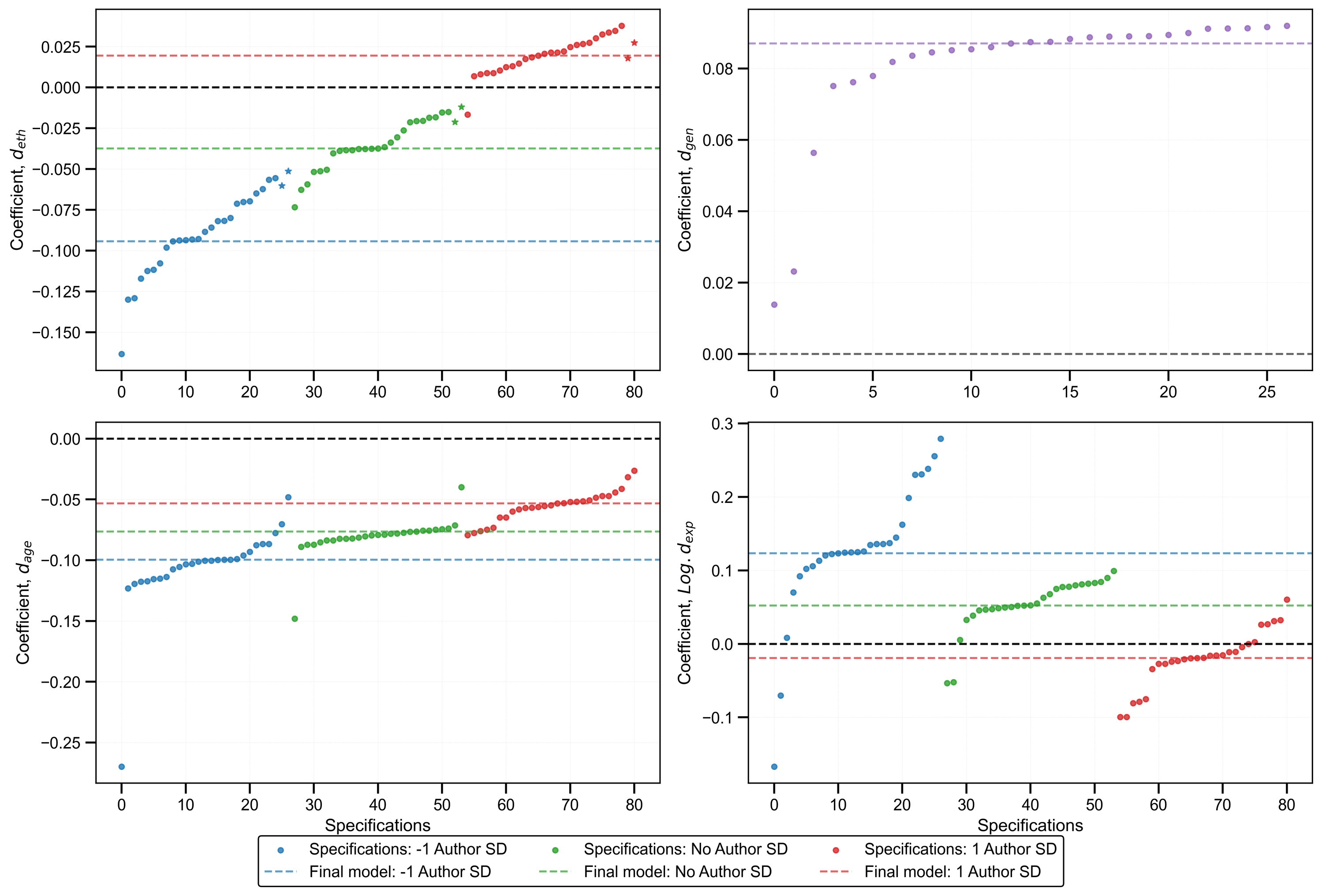

Supplement: S4 Fig — Each subplot displays the ordered array of marginal estimates (including their interaction with author count) for the diversity indices across all specifications, with the horizontal line marking the estimate for the observed data. For diversity indices dependent on author count, dx ∈ (eth, age, exp), the estimates total 81. In contrast, dx = (gen), which is not dependent on author count, totals 27. The asterisk * indicates non-significant specifications. (TIF) [file pone.0316890.s005.tif]

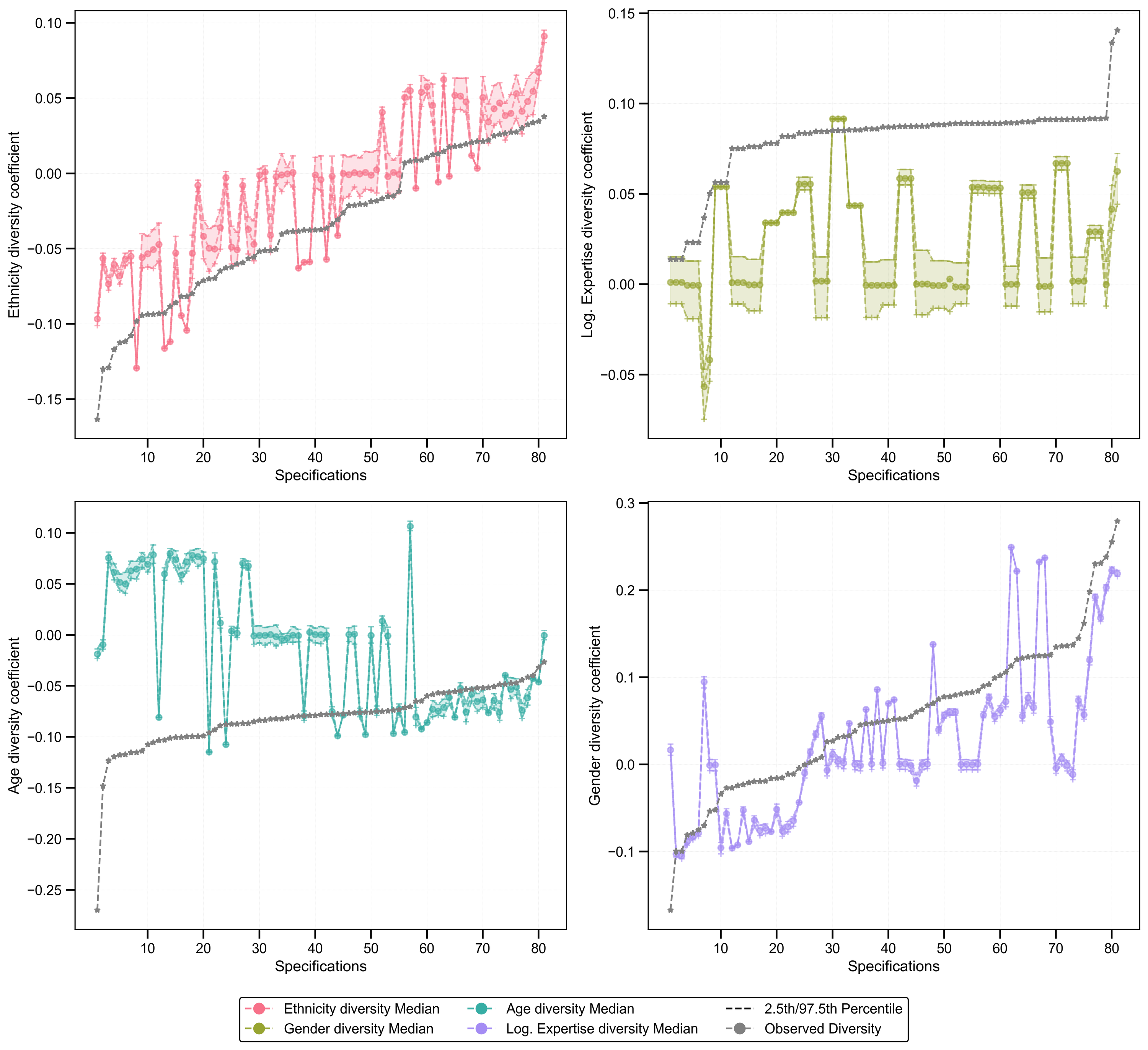

Supplement: S5 Fig — Each subplot displays the ordered estimates for the diversity indices across all specifications, comparing the observed and the expected under-the-null distribution. The expected curves are based on 50 shuffled samples where the key predictor, the diversity index value, is shuffled. All specifications are estimated on each shuffled sample, and the dashed lines depict the 2.5th, 50th, and 97.5th percentiles for each of these ordered estimates. The narrow confidence bands under the null for all diversity indices and the consistently low p-values indicate strong evidence for a robust and significant relationship for the observed data. (TIF) [file pone.0316890.s006.tif]
